# Mapping the emotional homunculus with fMRI

**DOI:** 10.1016/j.isci.2024.109985

**Published:** 2024-05-18

**Authors:** Michelle Giraud, Laura Zapparoli, Gianpaolo Basso, Marco Petilli, Eraldo Paulesu, Elena Nava

**Affiliations:** 1 Department of Psychology, University of Milano-Bicocca, Piazza dell’Ateneo Nuovo 1, 20126 Milan, Italy; 2 Psychology Department and NeuroMi, Milan Centre for Neuroscience, University of Milano-Bicocca, Milan, Italy; 3 fMRI Unit, IRCCS Istituto Ortopedico Galeazzi, Milan, Italy; 4 School of Medicine and Surgery, University of Milano-Bicocca, Piazza dell’Ateneo Nuovo 1, 20126 Milano, Italy

**Keywords:** neuroscience, cognitive neuroscience, behavioral neuroscience

## Abstract

Emotions are commonly associated with bodily sensations, e.g., *boiling with anger* when overwhelmed with rage. Studies have shown that emotions are related to specific body parts, suggesting that somatotopically organized cortical regions that commonly respond to somatosensory and motor experiences might be involved in the generation of emotions.

We used functional magnetic resonance imaging to investigate whether the subjective feelings of emotion are accompanied by the activation of somatotopically defined sensorimotor brain regions, thus aiming to reconstruct an “emotional homunculus.” By defining the convergence of the brain activation patterns evoked by self-generated emotions during scanning onto a sensorimotor map created on participants’ tactile and motor brain activity, we showed that all the evoked emotions activated parts of this sensorimotor map, yet with considerable overlap among different emotions. Although we could not find a highly specific segmentation of discrete emotions over sensorimotor regions, our results support an embodied experience of emotions.

## Highlights

• Observe to what extent the self-generated emotions map into the sensorimotor areas
• Participants performed an emotional recall task during fMRI scanning
• Sensorimotor maps are constructed from subjective tactile and motor stimulations
• Recalled emotions overlapped with the reconstructed sensorimotor maps

## Graphical abstract

**Figure:**
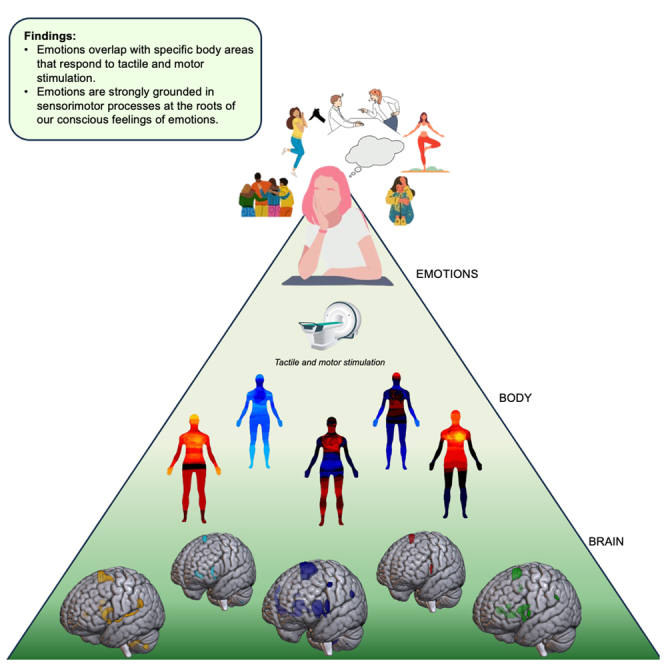

## Introduction

Body-emotion interactions have been explored for over a century: the James-Lange theory posited that our emotions are caused by changes in bodily sensations (“*I am shaking, that is why I am afraid*”) and thus placed the body at the origins of our conscious feelings. The Cannon-Bard theory claimed, on the contrary, that emotions and physical reactions co-occur, and similar physical reactions underlie different emotional states (“*My heart races because I am furious or because I am in love*”). Even the way we daily talk about emotions and feelings seems to invite us to think of emotions in embodied terms (e.g., “*it made my blood boil*”, “*her heart sank*,” “*her hair stood on end*”).^1^^,^^2^^,^^3^^,^^4^ On a conceptual level and using metaphors, emotions can be communicated verbally through literal somatic sensations or through words that refer to both literal and imaginative processes occurring within the body or with the body. The fact that we think, talk, and communicate emotions to others in an embodied fashion may provide some clues about the existence of a strong relationship between emotions and the body.

More recently, Damasio reinvigorated the James-Lange theory by showing that emotions can only be felt after the brain has registered the bodily changes accompanying them.^5^ Indeed, feelings are seen as mental experiences of body states, and their neural substrates can be found at all levels of the nervous system, from subcortical to cortical regions. In a pivotal study, Damasio and colleagues^6^ demonstrated that the process of feeling emotions requires the engagement of brain regions involved in the homeostasis of internal body states, such as the somatosensory cortices and upper brainstem nuclei. Based on the hypothesis that, in addition to the neutral sites already identified in the amygdala and orbitofrontal cortex, emotions also involve cortical and subcortical regions involved in representing and regulating internal body states (e.g., the insular cortex, the secondary somatosensory cortex, the anterior and posterior cingulate cortex, and brainstem tegmentum nuclei),^6^ 41 participants were asked to re-enact intense personal emotional episodes involving sadness, happiness, anger, and fear during positron emission tomography (PET) scanning. The re-enactment of such emotional experiences correlated with activity in the aforementioned brain regions, which is in line with modern views of *Embodied* *Cognition*, whereby the body actively participates in the construction of cognition.^1^^,^^7^^,^^8^^,^^9^^,^^10^^,^^11^^,^^12^^,^^13^

In this view, embodiment arises from the connection between the body, emotions, brain, and the environment,^14^ in which the body is no longer seen as a simple sensory-motor interface but becomes—jointly with the mind—an integrated biological system modulated by the experiences provided by homeostatic self-regulation interconnected with others and the environment.^15^ Hence, the embodiment is modulated not only by bodily experiences but also by affective experiences and internal body representation.^15^^,^^16^ In this sense, emotions are just as embodied as other cognitive systems and contribute to re-enacting sensorimotor experiences. Behavioral and neurophysiological evidence has corroborated the idea that emotions are strongly embodied^17^^,^^18^^,^^19^^,^^20^^,^^21^^,^^22^^,^^23^; for example, Nummenmaa and colleagues have shown that different emotional states are associated with topographically distinct and culturally universal body sensations,^24^^,^^25^^,^^26^ so that different body parts code different emotions. Indeed, Nummenmaa et al.^26^ found that most positive emotions are associated with sensations in the upper chest area; in contrast, sensations in the upper limbs are more prominent for emotions such as anger and fear, and sensations of decreased limb activity appear to be a hallmark of sadness. Even though these measurements are physiologically nonspecific and consist of self-reports, they reveal a far more active than hypothesized body participation in emotional processes. Neuroimaging studies have further supported this view by showing the direct involvement of somatosensory and motor systems in the perception and generation of emotions. Perceiving vocal and facial expressions of emotion triggers activity in the right somatosensory cortex, which correlates with subjective experience,^27^ and damage to this same region disrupts recognition of emotions from facial and vocal expressions.^28^^,^^29^ Importantly, electroencephalogram studies assessing visual evoked potentials (VEPs) and somatosensory evoked potentials (SEPs) have disentangled the role of these two sensory systems by revealing that only the somatosensory cortex is engaged in facial emotion recognition,^19^^,^^30^ providing neural evidence of embodiment of emotional expressions beyond the visual analysis of emotions.

Interestingly, an exploratory analysis has suggested that the somatosensory cortex can discriminate among discrete emotions^27^: it found that patterns of activations in the postcentral gyrus and the insula discriminated among perceived emotional categories, in line with the view that the emotions are (partly) reflected in the cortical representation of the body.^6^^,^^27^^,^^31^ Moreover, the relationship between emotions and the body appears to be bidirectional, in that emotions are not only embodied in the body but manipulating bodily signals (e.g., physiological or postural) can influence the perception and generation of emotions.

Recent studies have shown that false physiological feedback of evoked or tonic bodily responses can alter emotional attributions (e.g., by providing false feedback of increased heart rate). It has been observed that perceived emotional intensity/salience of neutral faces increases when accompanied by false feedback of increased heart rate,^32^ and, the more accurately participants can track heart rate, the stronger the observed link between heart rate changes and subjective ratings of arousal (but not valence) of emotional images.^33^ For example, the processing of brief fear stimuli is selectively gated by their timing in relation to individual heartbeats: fearful faces were detected more easily and rated as more intense at systole than at diastole.^34^

Even some artificial manipulation of organ activity can induce emotions; for instance, intravenous administration of cholecystokinin can provoke panic attacks.^35^^,^^36^

More generally, theories of bodily feedback shed light on this bidirectional emotion-body relationship, suggesting that manipulating facial expressions and body postures can influence emotional reactions to stimuli and physiological responses (e.g., heart rate and skin conductance). Moreover, the manipulation of bodily states can influence cognitive processes, such as the speed with which individuals read emotional content and categorize emotional information and the extent to which they determine emotional information as threatening.^37^ All these bidirectional influences more generally reveal the tight link between emotional and bodily experiences and the need to explore the neural underpinnings of such relationships.

### Aim and predictions of the study

To date, no study has assessed the presence of topographically distinct body maps at the brain level while discrete emotions take form. In fact, despite the evidence implicating the somatosensory system in emotional processes, it is still unclear whether the formation of discrete emotions activates brain regions specifically related to body parts principally involved in the perception of tactile and motor events. Therefore, the main aim of this study was to observe how activity in the sensorimotor cortex relates to subjective generation of emotions, with the hypothesis that, if emotions are indeed felt in the body, their perception should correspond to the activation of specific portions of sensorimotor areas corresponding to the part of the body associated with that emotion. Specifically, the aim was 2-fold: first, we aimed to investigate the relationship between the emergence of the subjective feeling of emotion and the appearance of perceived sensations in the body from a neural perspective; second, we tried to reconstruct an “emotional homunculus” with topographically distinct fingerprints according to the emotions and the bodily sensations experienced. Furthermore, we aimed to observe a possible similarity between the neural and the behavioral data (as assessed from drawn silhouettes in which participants indicated the body part associated with a given emotion) using representational similarity analysis (RSA).

Based on the hypothesis that emotions are embodied and can be represented through partially extended body sensation maps, as suggested by behavioral studies,^24^^,^^26^^,^^38^^,^^39^ we used functional magnetic resonance imaging (fMRI) during the online generation of five emotions (happiness, sadness, fear, anger, serenity) through the enactment of emotionally salient autobiographical episodes. In addition, we used a tactile and motor localization task to create extensive and personalized sensorimotor maps. These sensorimotor maps were then applied as a guide for exploring the fMRI patterns derived from the emotional recall task. This analysis allowed us to investigate whether the retrieval of emotions overlapped with specific body sensation maps. We used maps of both somatosensory and motor activity, given the evidence that perceiving and thinking about emotions not only are a mere perceptual and somatic-visceral matter but also involve a motor recall of the emotion itself.^31^ Neuroimaging studies have shown that information about each body part is based on multiple cortical representations not limited to a highly selective region within a topographic map (i.e., Penfield’s homunculus in areas S1 and M1)^40^^,^^41^^,^^42^^,^^43^; rather it is widely distributed, in line with what is observed in non-human primates.^44^^,^^45^^,^^46^^,^^47^ In general, the sensory, motor, and affective neural populations are highly interconnected, and their activation promotes a multimodal and integrated experience of the emotional event. The re-enactment of a salient emotional event is termed “embodied” because the past event is re-experienced first at the level of the involved sensorimotor systems, as if the individual were present in the same situation, emotional state, or thought object.^31^

In line with the previous literature on embodiment studies, we expected to find a convergence between the sensorimotor maps reconstructed during the somatosensory and motor localizer tasks and the recall of autobiographical emotional episodes. In particular, we expected three different possible outcomes: first, to find the presence of topographically distinct body fingerprints as discrete emotions take shape; second, to be able to reconstruct an “emotional homunculus” similar to the somatosensory and motor homunculus already known; and third, to observe a possible similarity between the bodily sensations participants associated graphically with a specific emotion (i.e., using pen-and-paper silhouettes) and the corresponding neural activation. We had no *a priori* expectations that we could find a significant result for all outcomes, but what is important here is that our experimental design allowed us to compare the three possibilities analytically.

## Results

### Behavioral results

#### The pre- and post-scan silhouettes analysis

The correlation between the pre- and post-scan silhouettes was highly significant (r = 0.825, *p* < 0.001), suggesting intra-individual consistency in the distribution of bodily sensations related to emotions, in turn revealing no perturbing effects on the scanning per se on such perceptions.

#### The subjective experience of emotions: Single-subject analysis on participants’ ratings

Behavioral ratings confirmed that the audio-recorded personal episodes used in the experiment induced the selected emotions in participants: the intensity and vividness of the elicited emotions were higher in comparison to their respective neutral episodes (intensity: all *p* < 0.001; all BF (Bayes Factor) > 756.091; vividness: all *p* < 0.022; all BF > 2.90), except for vividness during anger recall, which did differ marginally from its neutral control (*p* = 0.053, BF = 1.21) (for further details see Table S1).

#### The subjective experience of emotion: Single-subject analysis on self-report body silhouettes

**Figure 1:**
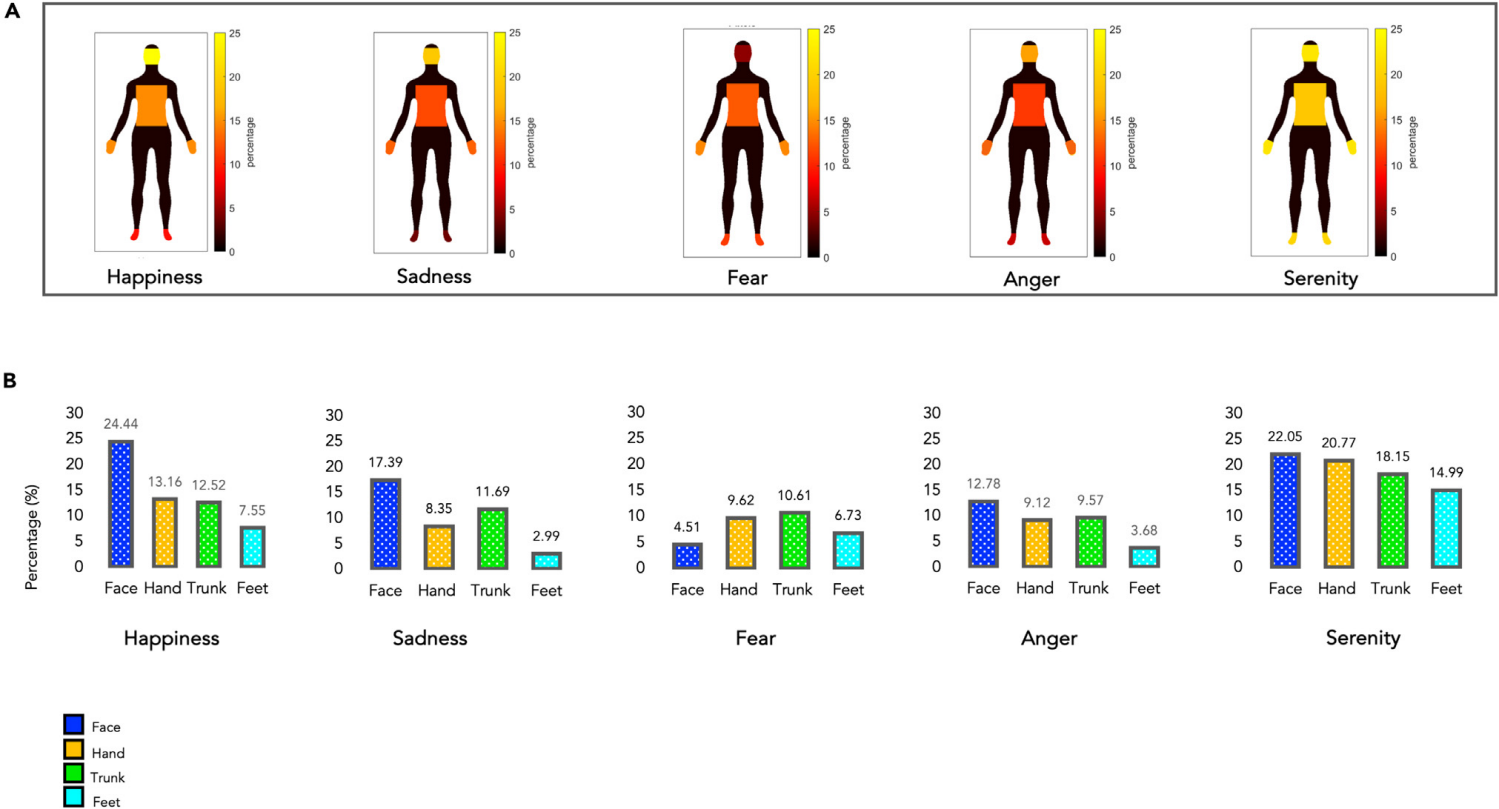
The subjective experience of emotions: single-subject analysis on self-reports silhouettes (A) Emotional homunculi based on subjective self-reports. Digitization of the colored silhouettes by the participants. Each silhouette was divided into four discrete body districts, the same ones used during the functional imaging tasks. Within each body segment, the percentage of colored pixels in that specific body part was calculated. (B) Graphical representation (in percentages) of the distribution of emotions across discrete body parts (i.e., face, hands, trunk, feet; Bayesian one-sample t test all BF > 2.75, except sadness on feet [BF = 0.55] and anger on feet [BF = 0.61]).

**Figure 2:**
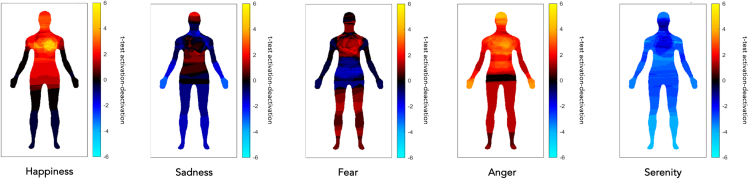
The digitalized subject-wise body-emotion maps Result of the digitalized subject-wise bodily sensations maps. Each map was obtained by subtracting the deactivation map from the activation map.^16^ Warm colors indicate increasing activation (e.g., feeling muscle movements, the temperature increasing, or increasing heartbeat, etc.), while cool colors represent decreasing activation (e.g., feeling relaxed muscle, freezing sensation, decreasing heartbeat, etc.). The color bar shows the t-statistic range.

Participants’ self-reports re-coded into colored body silhouettes showed that most emotions were associated with bodily experiences in all four body districts tested. Indeed, Bayesian one-sample t tests revealed that all emotions activated all body parts (all *p* < 0.019, all BF > 2.75), except sadness on the feet (*p* = 0.148, BF = 0.55) and anger on the feet (*p* = 0.129, BF = 0.61) (see Figure 1; Table 1 and Figure 2).

**Table 1:** Total percentage of activation of a specific body segment during the re-enactment of emotion

	Face (%)	Hand (%)	Trunk (%)	Feet (%)
Happiness	24.44	13.16	12.52	7.55
Sadness	17.39	8.35	11.69	2.99
Fear	4.51	9.62	10.61	6.73
Anger	12.78	9.12	9.57	3.68
Serenity	22.05	20.77	18.15	14.99

Figure S1 illustrates a comparison with Nummenmaa et al. (2014) data showing some similarities, despite the wide differences between the two datasets (e.g., somministration method and sample size). From a visual inspection of the two types of silhouettes, we can observe that happiness shows significant “activation” hotspots (i.e., yellow and red) in the face and trunk, such as sadness shows “activation” hotspots in the face and trunk and “deactivation” hotspots (i.e., blue and light blue) in the upper and lower limbs. Unfortunately, it was impossible to test the goodness of our replication because the raw data from Nummenmaa et al. (2014) study were not available.

### fMRI results

#### Somatosensory and motor localizer tasks

##### Brain regions with neurons mapping all the body segments investigated: Conjunction map of motor execution and tactile stimulation

**Figure 3:**
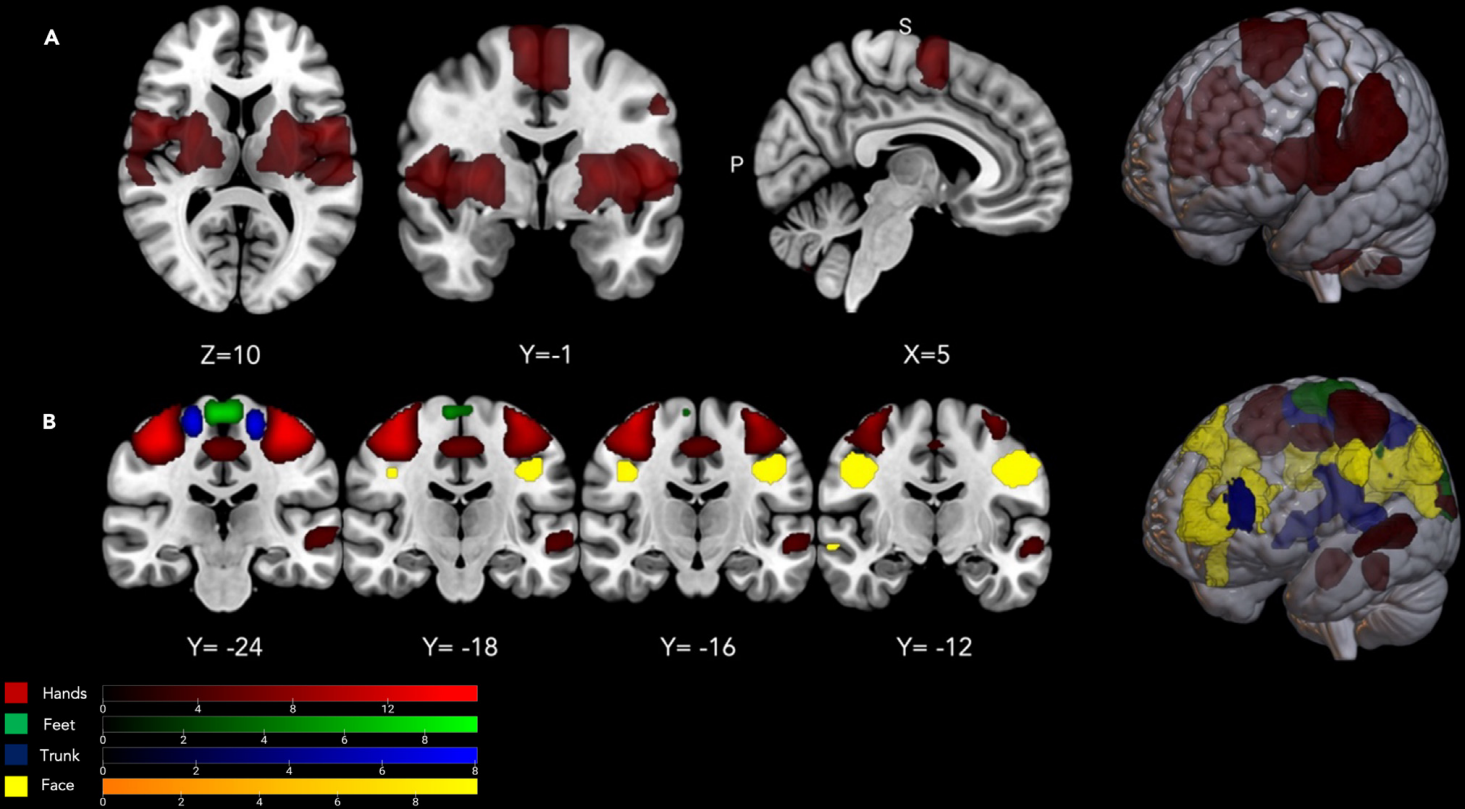
Brain regions with neurons mapping the whole body or specific-body-segments effects (A) Brain regions that resulted significantly active for the conjunction of Motor and Tactile localizer tasks for all body segments. All the data are reported by applying the same statistical threshold reported in the tables and discussed in the text (puncorr < 0.001 at the voxel level and pFWER-corr < 0.05 at the cluster level). (B) Brain regions that resulted significantly active for the contrast “Face movements & Face tactile stimulation > all the other body segments movements & tactile stimulation,” “Hands movements & Hand tactile stimulation > all the other body segments movements & tactile stimulation,” “Trunk movements & Trunk tactile stimulation > all the other body segments movements & tactile stimulation,” “Feet movements & Feet tactile stimulation > all the other body segments movements & tactile stimulation.” All the data are reported by applying the same statistical threshold reported in the tables and discussed in the text (puncorr < 0.001 at the voxel level and pFWER-corr < 0.05 at the cluster level).

**Figure 4:**
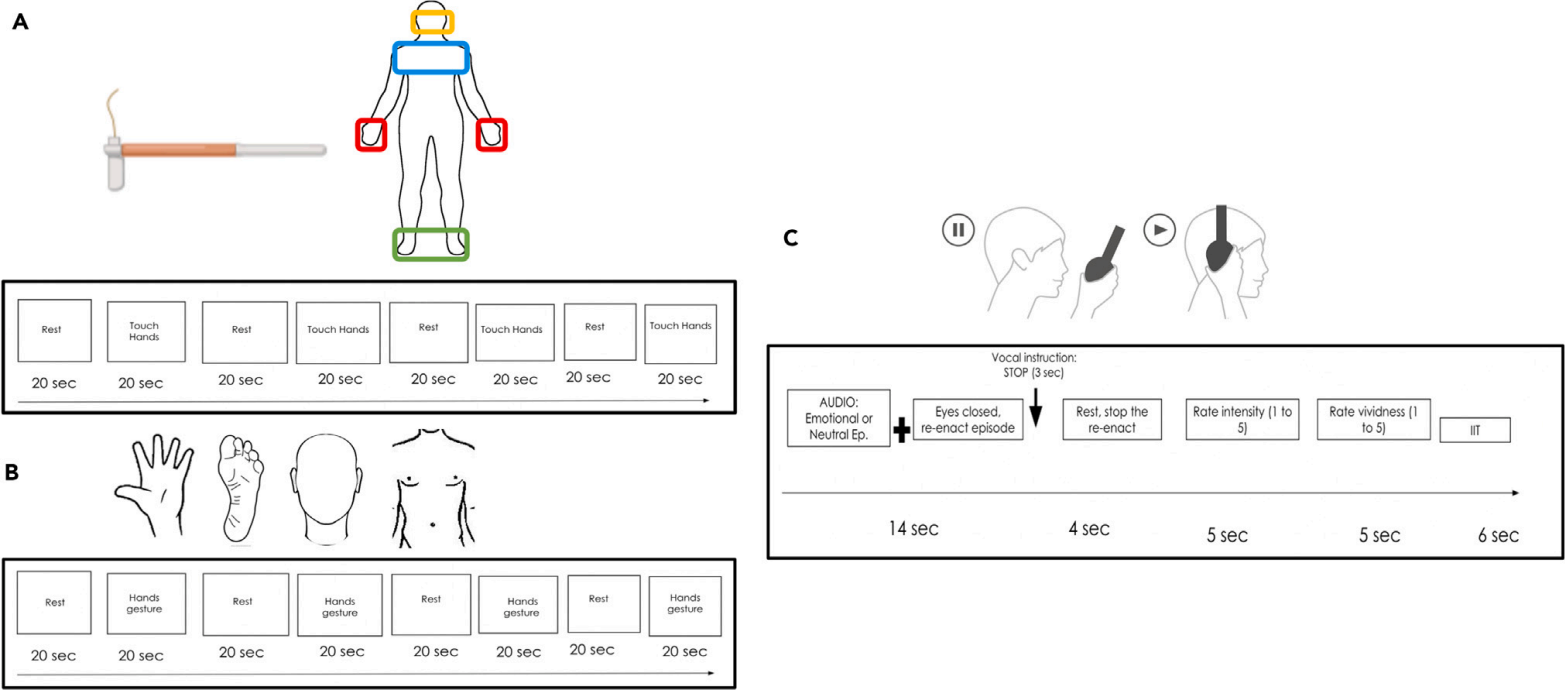
Experimental design (A) Somatosensory functional localizer task procedure. Each participant was bilaterally stimulated using Von Frey filaments 60 gr. (B) Motor functional localizer task procedure. Each participant performed specific movements using their hands, feet, trunk, and face. (C) Emotional recall task procedure. Each participant heard through headphones 14s of emotional autobiographical episodes followed by neutral autobiographical episodes in random order.

We shall call these “whole-body” brain maps, as opposed to the more specific brain maps described in the following. As expected, the execution of movements with different body segments and their tactile stimulation activated a large neural network involving premotor, motor, and somatosensory cortical and subcortical brain regions bilaterally, including secondary somatosensory regions like, for example, area SII (secondary somatosensory area), ventral premotor cortex, the insulae, etc. (see Figure 3A; for further details, see Table S2, and Figures 4A and 4B). All these regions contain neurons with broad somatosensory receptive fields, often covering both sides of the body.^48^^,^^49^^,^^50^

##### Specific-body-segments effects: Body-segment-specific maps

Each body part’s movement or the bilateral tactile stimulation was associated with a somatotopically organized bilateral activity of premotor, motor, and somatosensory brain regions (see Figure 3B; for further details, see Table S3 and Figures 4A and 4B), replicating the regional distribution of Penfield’s homunculus around the central sulcus. These are illustrated in Figure 3B, from lateral ventral to dorsomedial areas: the face area (in yellow), the hand area (in red), the trunk area (in blue). and the feet area (in green).

#### Emotional recall task

##### Brain activity evoked by the emotional recall task: Main effect of the emotional recall

**Figure 5:**
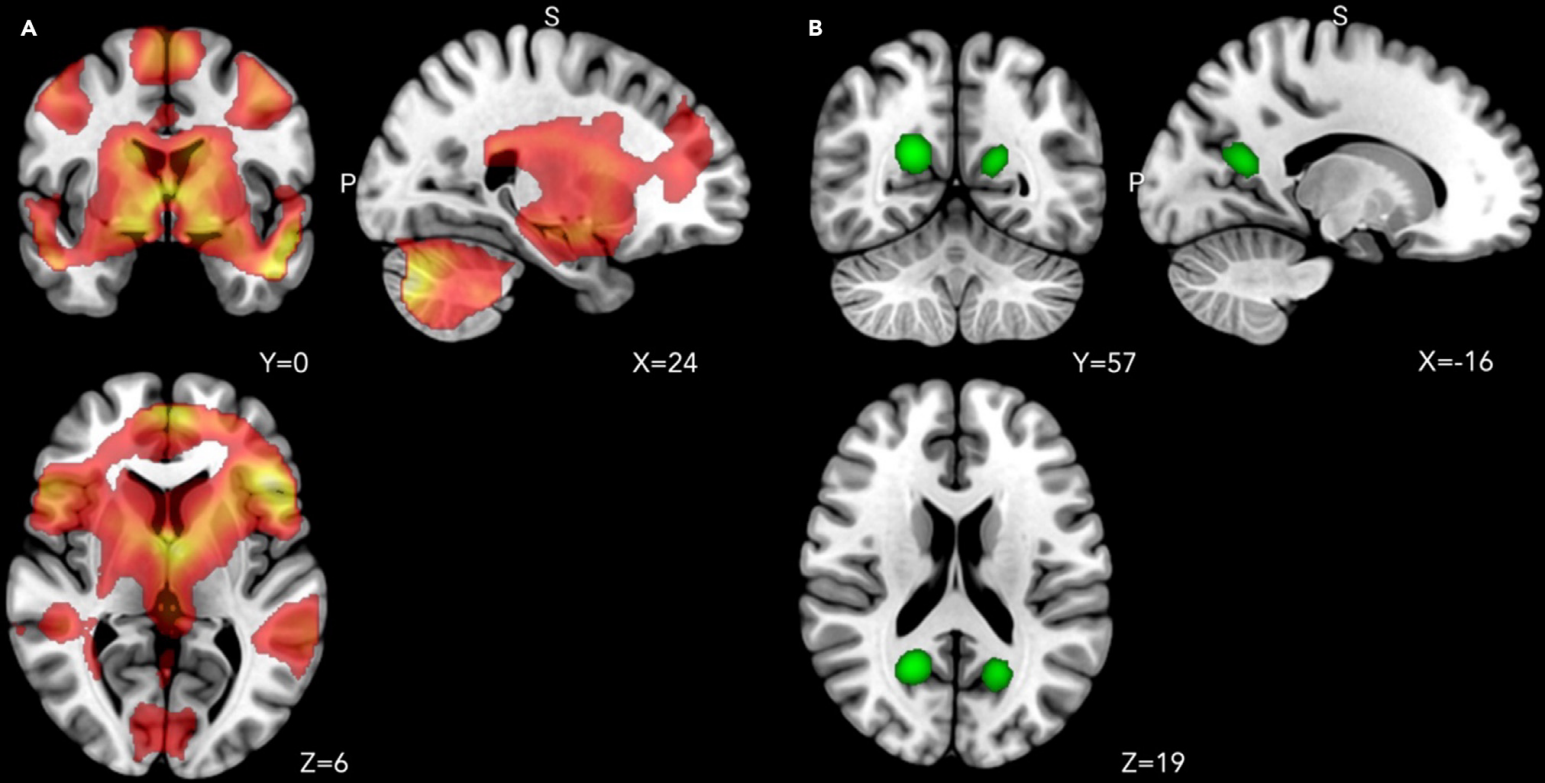
Brain activity evoked by the emotional recall task (A) Brain regions that resulted significantly active for the contrast “Emotional episodes > Neutral episodes.” All the data are reported by applying the same statistical threshold reported in the tables and discussed in the text (puncorr < 0.001 at the voxel level and pFWER-corr < 0.05 at the cluster level). (B) Brain regions that resulted significantly active for the contrast “Neutral episodes > Emotional episodes.” All the data are reported by applying the same statistical threshold reported in the tables and discussed in the text (puncorr < 0.001 at the voxel level and pFWER-corr < 0.05 at the cluster level).

The active recall of emotional autobiographical episodes, compared with the recall of neutral episodes, was associated with the activity of a wide frontotemporal network, including bilaterally the pre-SMA (supplementary motor area), the amygdala, and the hippocampus. Further activations were found at the level of the left middle temporal gyrus, the left thalamus, the left amygdala, and the right cerebellum (see Figure 5A; for further details see Table S4 and Figure 4C).

The opposite contrast (neutral recall > emotional recall) revealed the activation of occipital brain regions, including the bilateral cuneus and the middle occipital gyrus (see Figure 5B; for further details, see Table S5).

##### The intersection of emotional recall on whole-body brain areas divided in tactile and motor behavior

**Figure 6:**
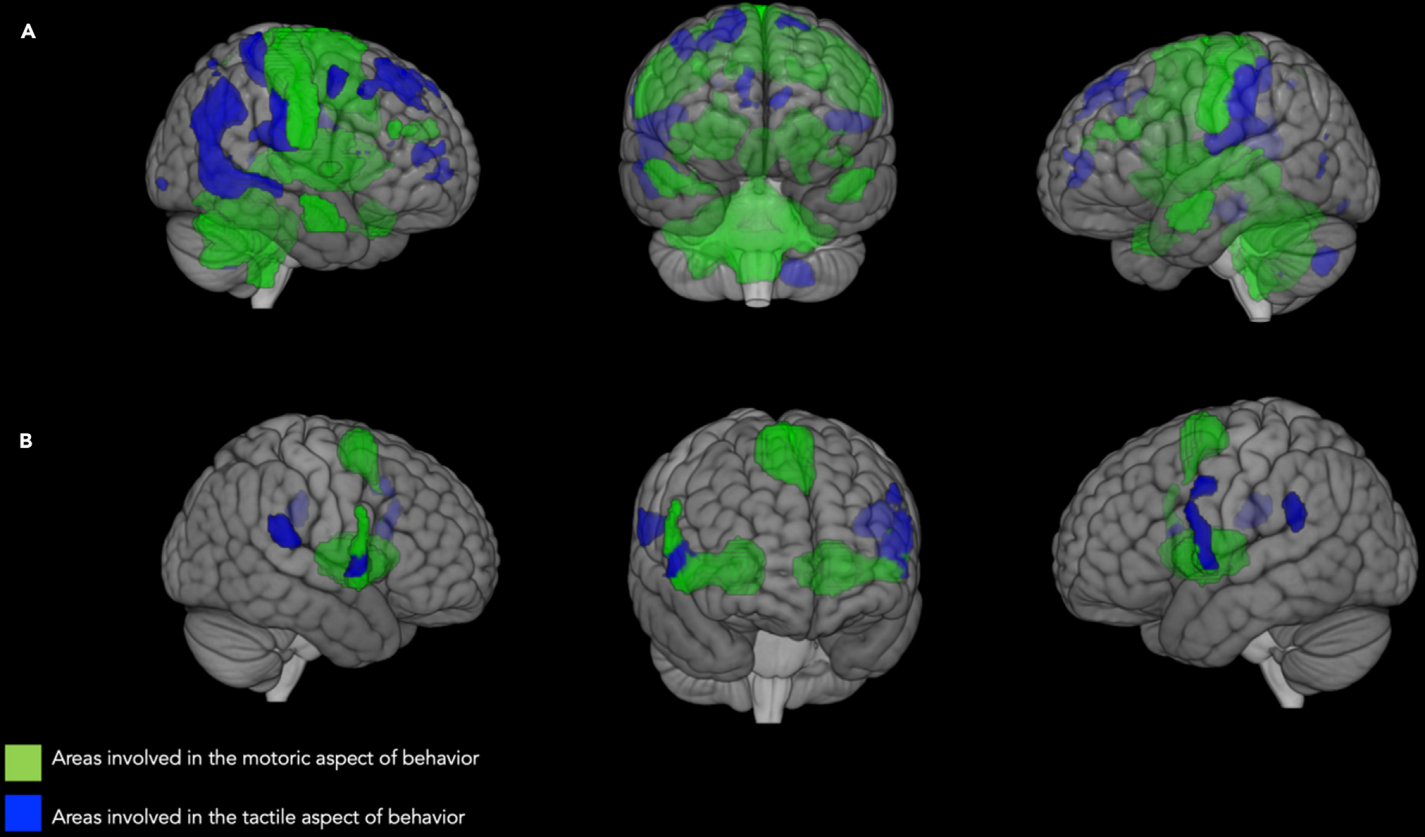
The intersection of emotional recall on whole-body brain areas (A) Intersection areas of individual body parts involved in the motor and tactile aspects, defined as a single conjunction effect. Here, a distinction is made between regions more active for the motor localizer task (in green) or for the tactile localizer task (in blue). All the data are reported by applying the same statistical threshold reported in the tables and discussed in the text (puncorr < 0.001 at the voxel level and pFWER-corr < 0.05 at the cluster level). (B) Anatomical overlap of the main effect of the emotional recall task and regions with neurons responding to all body segments tested, a distinction is made between regions more active for the motor localizer task (in green) or for the tactile localizer task (in blue). All the data are reported by applying the same statistical threshold reported in the tables and discussed in the text (puncorr < 0.001 at the voxel level and pFWER-corr < 0.05 at the cluster level).

First, we created two separate maps of the whole body: one for the motor aspects (e.g., motor >touch for face; motor >touch for hand, etc.) and one for the tactile aspects (e.g., touch >motor for feet, touch >motor for trunk, etc.) (see Figure 6A). Then, these two maps were used as masks for the main effect of the emotional recall task. The emotional recall shows a conspicuous overlap with anatomical areas involved in motor aspects of behavior (green areas), including the ventral premotor cortex, SMA, and somatosensory regions that map the entire body in a limited space (blue areas) (See Figure 6B).

##### Discrete emotions and the whole-body sensorimotor map

**Figure 7:**
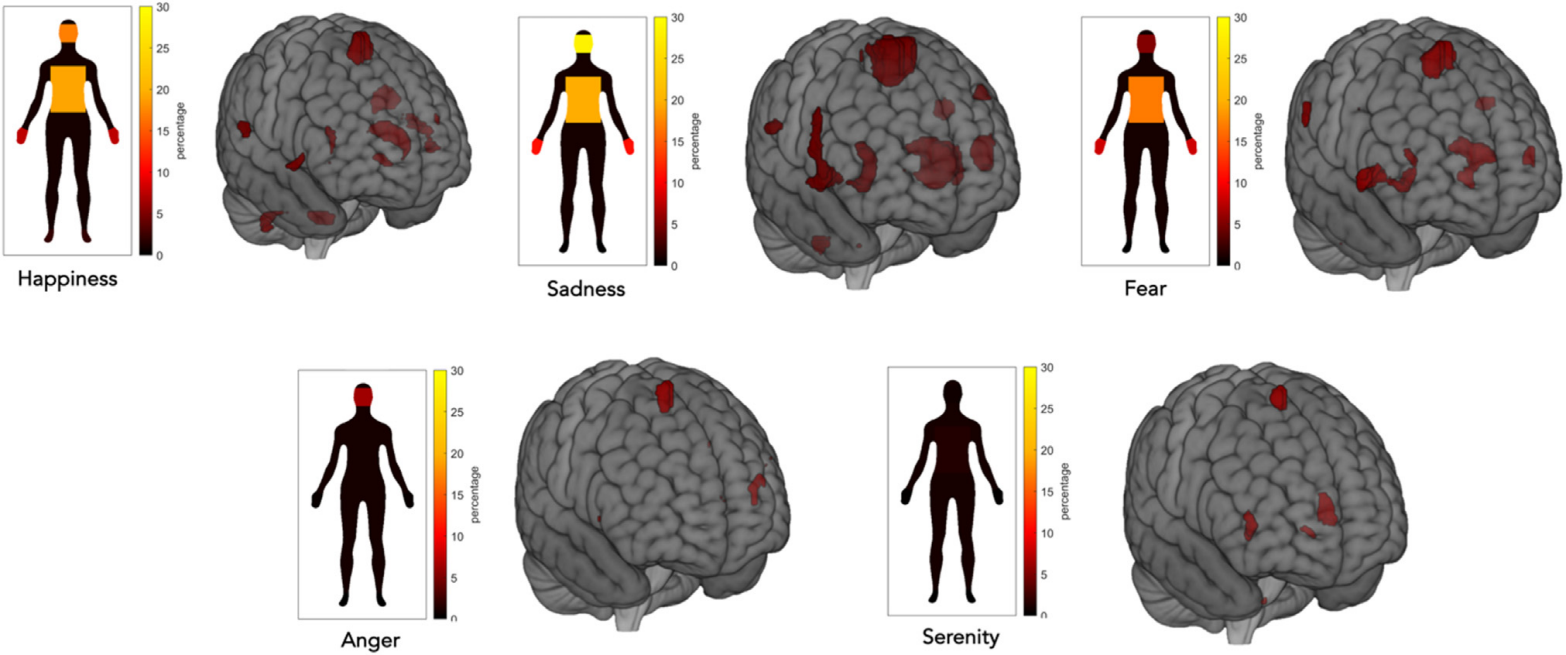
Discrete emotions and the whole-body sensorimotor map Overlay of the five discrete emotion maps onto the sensorimotor whole-body map. Note that all emotions significantly overlapped with the conjunction map of the localizer scans. All the data are reported by applying the same statistical threshold reported in the tables and discussed in the text (puncorr < 0.001 at the voxel level and pFWER-corr < 0.05 at the cluster level).

The brain activity evoked by recalling specific emotions differently overlapped with the sensorimotor conjunction map described in the first part of the paragraph entitled "Somatosensory and motor localizer tasks" All the emotions explored (happiness, sadness, fear, anger, serenity) activated parts of these sensorimotor maps (see Figure 7; Table 2; for further information, see Table S6).

**Table 2:** Convergence (in percentage) of brain activation patterns evoked by self-generated emotional experiences with the somatosensory-motor map scan created based on participants’ tactile and motor brain activity

	Sensorimotor maps (%)
Happiness	8.22
Sadness	19.71
Fear	6.44
Anger	0.76
Serenity	1.63

##### Discrete emotions and body-segment-specific maps

**Figure 8:**
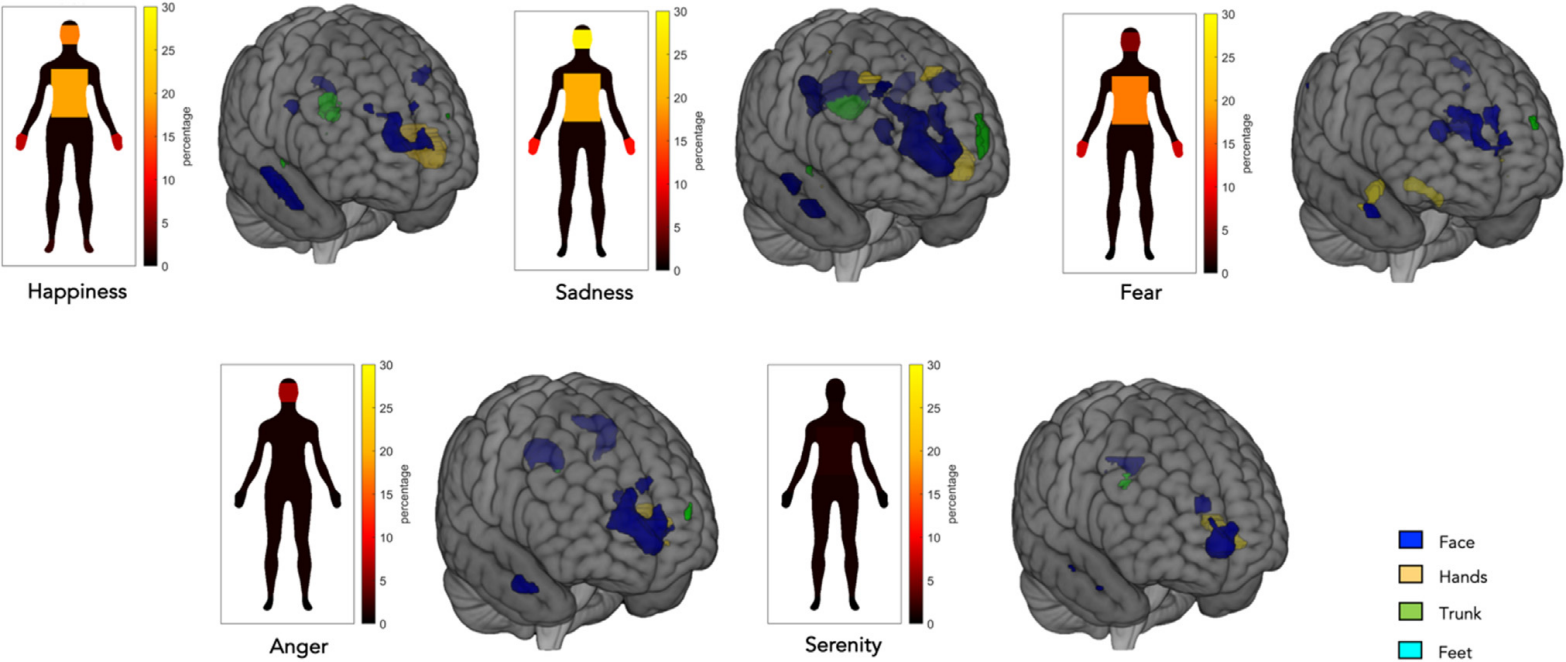
Discrete emotions and body-segments-specific maps Overlay of the five discrete emotion maps onto the discrete body-segments maps based on group-level analyses. Note that all emotions significantly overlapped with the specific four body-segment maps, except for feet in happiness and in fear, which showed no selective voxels activation. All the data are reported by applying the same statistical threshold reported in the tables and discussed in the text (puncorr < 0.001 at the voxel level and pFWER-corr < 0.05 at the cluster level).

The brain activity evoked by the recall of specific emotions differently overlapped with specific-body-segment fMRI maps described in the second part of the paragraph entitled "Somatosenory and motor localizer tasks". All four body segments (i.e., face, hands, trunk, and feet) showed a response for the five emotions, except for feet in happiness and in fear, which showed no selective voxels activation (see Figure 8; Table 3; for further details, see Tables S7, S8, S9, S10, and S11). In particular, we can observe that specific emotions activated different brain regions related to sensorimotor representations of distinct body segments (see Table 4).

**Table 3:** Convergence (in percentage) of brain activation patterns evoked by self-generated emotional experiences with the discrete body parts based on participants’ tactile and motor brain activity

	Face (%)	Hand (%)	Trunk (%)	Feet (%)
Happiness	3.82	9.81	2.39	–
Sadness	17.01	8.87	3.49	6.20
Fear	3.64	2.83	0.19	–
Anger	9.28	3.49	0.29	2.34
Serenity	2.89	2.17	0.20	0.24

**Table 4:** Contingency table, emotion regions across all body segment

	Happiness	Serenity	Fear	Anger	Sadness
Frontal lobe	∗		∗	∗	∗
Temporal lobe	∗		∗		
SMA	∗	∗	∗	∗	∗
Rolandic operculum	∗				
Thalamus	∗		∗		∗
Insula	∗			∗	∗
Putamen	∗	∗	∗		∗
Cerebellum	∗	∗	∗		∗

##### The subjective feeling of emotions: Single-subject level analysis on brain activations

**Figure 9:**
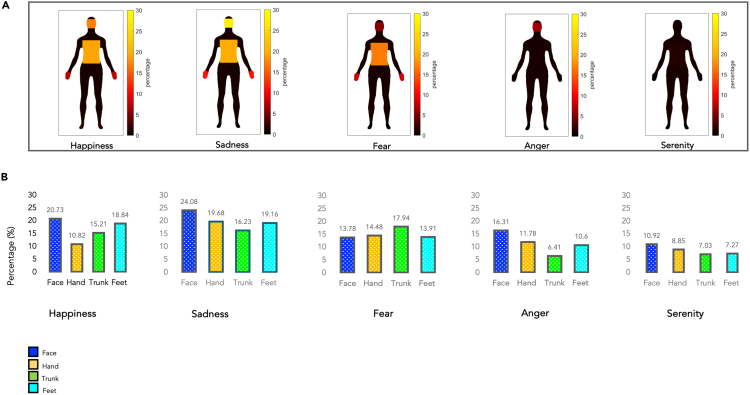
The subjective experience of emotions: single-level analysis on brain activations (A) Emotional homunculi based on brain activation at single-subject level analysis. Each silhouette was divided into four discrete body districts, the same ones used during the functional imaging tasks. Within each body segment, the percentage of brain activations in that specific body part was calculated. (B) Graphical representation (in percentages) of the distribution of emotions across discrete body parts (i.e., face, hands, trunk, feet; Bayesian one-sample t test all BF > 7.00, except for serenity in feet [BF = 1.69] and trunk in feet [BF = 1.35]). The voxel-wise threshold applied to the statistical maps before cluster correction was *p* < 0.05 uncorrected to maximize the chance of detecting effects in the less sensitive single-subject fixed-effect analyses.

For each participant, we also calculated emotion-specific patterns of activations by masking each effect on the body-specific group-level sensorimotor maps (see Figure 9; Table 5).

**Table 5:** Total percentage of activation of a specific body part during the re-enactment of emotion under scanning

	Face (%)	Hand (%)	Trunk (%)	Feet (%)
Happiness	20.73	10.82	15.21	18.84
Sadness	24.08	19.68	16.23	19.16
Fear	13.78	14.48	17.94	13.91
Anger	16.31	11.78	6.41	10.60
Serenity	10.92	8.85	7.03	7.27

This analysis showed that all emotions activate the body segments considered for this study, although no body segment seems to be uniquely important for the emergence of a single emotion. Through (Bayesian) one-sample t tests, we assessed that all emotions activated all body parts (all *p* < 0.011, BF > 7.00), except serenity in feet (*p* = 0.052, BF = 1.69) and trunk (*p* = 0.057, BF = 1.35). In particular, the face is the body segment most systematically involved in feeling most emotions.

##### Congruence between body distribution of emotions as depicted by self-reports and fMRI

We did not find a similarity between the self-report silhouettes and the neural recall emotion; an RSA of the data failed to show systematic associations.

We used the RSA to assess the correspondence between brain activation patterns recorded during the fMRI emotion recall task and subjective feelings by each participant at the end of the experiment during the paper-and-pencil emBODY Task. We calculated similarity matrices between behavioral data (i.e., number of pixels colored for each emotion on each body part of the silhouettes) and fMRI data (i.e., number of voxels activated for each emotion, overlapped on each body-segment activation map).

The RSA is a computational technique that uses pairwise comparisons of stimuli to reveal their representation in higher-order space.^51^^,^^52^ Correlations between voxel and pixel matrices were computed for each participant individually and then tested against zero across participants using t tests. The t test on the coefficients of correlation was not significant (*p* = 0.20). No statistical similarity was found between the neuroimaging data (i.e., voxels) and the behavioral data derived from the participants' colored silhouettes (i.e., pixels), as can already be seen from the visual inspection of Figure S2.

**Figure 10:**
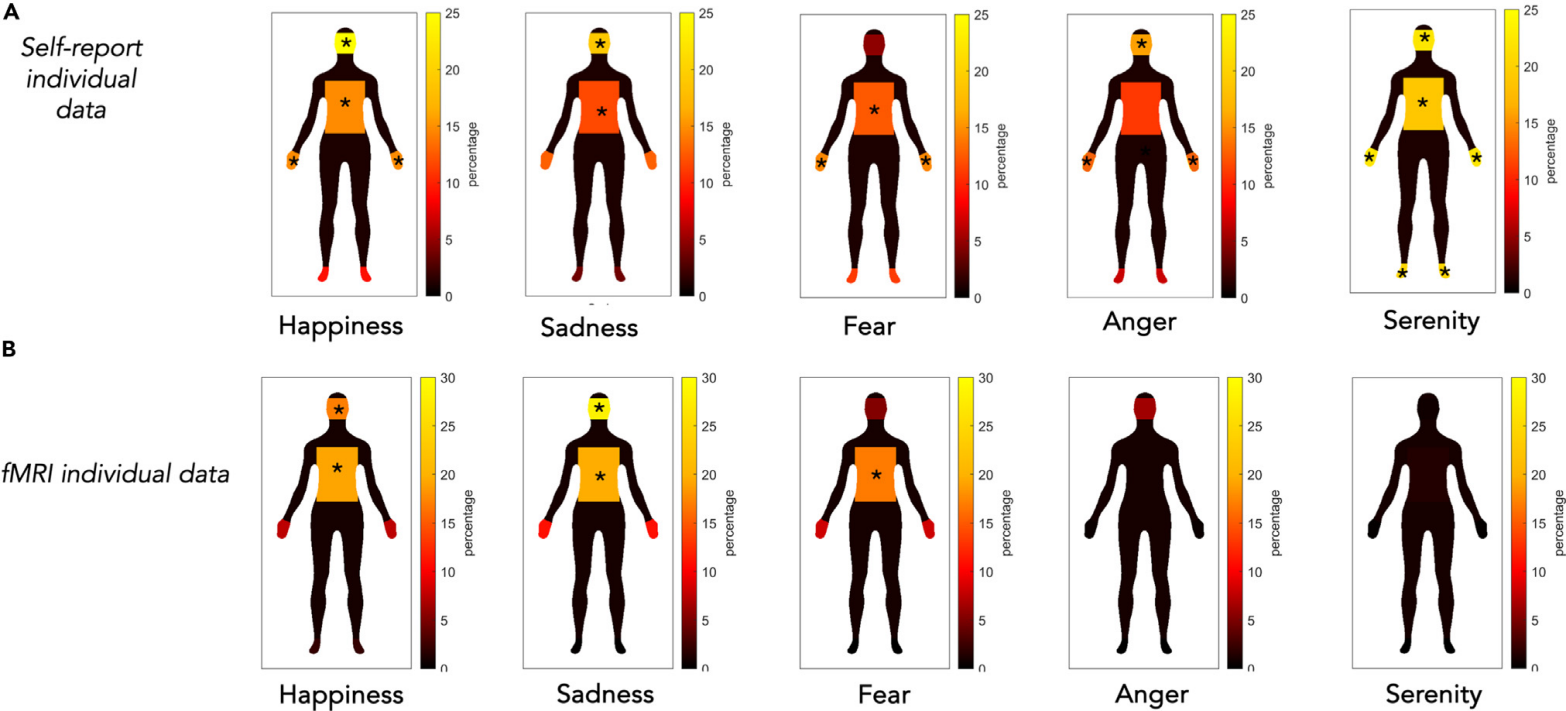
Congruence between body distribution of emotions as depicted by self-reports and fMRI The comparison between the silhouettes created by participants’ self-reports (i.e., digitized from the emBODY pen-and-paper task) and the silhouettes created by individual participants’ brain activation at single-subject level analysis. Both figures were created by considering the percentage of active pixels or voxels on the total surface area of a specific body parts (i.e., face, hands, trunk, and feet). The asterisks indicate hotspots, i.e., defined as body parts with maximal intensity. (A) Digitalized silhouettes from self-report individual data. (B) Digitalized silhouettes based on brain activation at single-subject level analysis (e.g., fMRI individual data).

Nevertheless, we qualitatively observed whether there was a potential visual similarity between behavioral and neural data. Using the same method employed for the emBODY silhouettes, we extracted and calculated the percentage of voxels active during the emotional recall task within each body part. This gave us the total percentage of activation of a specific body segment during the re-enactment of the emotion. Then, we obtained two new digitized silhouettes: A) one silhouette that visually represented the distribution of colored pixels within each body segments (i.e., four) for the five emotions and B) one silhouette that visually represented the distribution of neural activations within each body segment (i.e., four) for the five emotions (see Figure 10).

The emotional homunculi summarized in Figure 10 describe the cumulative congruence and variability of the distribution of the self-reports (see Figure 10A) and brain activations (see Figure 10B) on emotion-specific body sensations. We assumed that the fMRI signals should increase for any kind of bodily feeling; accordingly, we assumed that any change of bodily sensation from a virtual zero should be mapped by fMRI local activation. Exploration of these two types of silhouettes showed that, while no body segment seems to be uniquely important for classifying a single emotion for at least three emotions (i.e., happiness, sadness, and fear), the same body parts were hotspots, defined as body parts with maximal intensity, in both kinds of maps; on the other hand, for anger and serenity, no such correspondence can be observed (see Figure 10; Table 6).

**Table 6:** Hotspots shared between silhouettes created by self-reports and silhouettes created by brain activation

Emotions	Face		Hand		Trunk		Feet	
	Self-report	fMRI data	Self-report	fMRI data	Self-report	fMRI data	Self-report	fMRI data
Happiness	+	+	+	-	+	+	-	-
Sadness	+	+	-	-	+	+	-	-
Fear	-	-	+	-	+	+	-	-
Anger	+	-	+	-	-	-	-	-
Serenity	+	-	+	-	+	-	+	-

## Discussion

### Embodied feeling of emotions

Consistent with current theories of embodied emotions, in this study we attempted to find neural emotional fingerprints across motor and tactile areas to assess the direct involvement of the sensorimotor system in the generation of emotional experiences.

Participants were scanned during the active re-enactment of personal autobiographical emotional episodes of five discrete emotions (i.e., happiness, serenity, anger, fear, sadness) and neutral control episodes, and we examined whether the process of feeling emotions requires the participation of brain regions involved in mapping internal body representations and their movements, typically processed across somatosensory and motor areas. Inspired by the PET study by Damasio and colleagues,^6^ in which they effectively used the recall of emotional and neutral autobiographical episodes to induce emotional states, we tried to use stimuli that were more suitable for eliciting and experiencing subjective emotions,^53^^,^^54^^,^^55^ rather than using stimuli that might have made the emotional experience less personal and potentially less intense (e.g., visual stimuli or movies).

Our data reveal that the recall of emotions involves both tactile and motor experiences to be felt. Indeed, brain activation associated with the re-enactment of all five emotions overlapped with the neural activation of the map constructed on the participants’ joined tactile and motor activity across different body parts (i.e., faces, hands, trunk, and feet), demonstrating the strong link between discrete emotions and sensorimotor experiences.

In general, observing the five emotions together significantly overlapped with brain systems involved in motoric aspects of behavior, including the ventral premotor cortex, the SMA, and somatosensory regions that map the entire body in a limited space. This might shed light on a more central role of the sensorimotor system in various high-level cognitive processes necessary for interacting with the world.^8^^,^^9^^,^^10^^,^^56^ In particular, greater involvement of the motor system areas than the somatosensory system is observed in recalling emotional episodes, probably due to the very nature of the emotional recall task, in which participants were asked to recall themselves in reliving an emotion. Tasks using mental imagery (e.g., a motor imagery task) involve the activation of several sensorimotor areas similar to those that would be activated in correspondence with real movement, including the dorsal premotor cortex (dPMC) and primary somatosensory cortex (S1).^57^

### Discrete emotions overlap with sensorimotor map

Specifically, we found that sadness seems to be the most strongly represented emotion in the conjunction map (i.e., 19.71%), activating different brain areas such as the SMA, the insula, the ventral-lateral thalamus, and the inferior frontal gyrus (for further details, see Table S6 in the supplemental information). The fact that sadness activates the higher portions of the conjunction map may depend, on the one hand, on the nature of the emotion itself and, on the other hand, on the task itself—the autobiographical recall—that has been described as the most effective in recalling this emotion.^58^ Indeed, sadness is a commonly experienced emotion in everyone’s life that impacts the body and mind and whose subjective experience is uniquely represented by functional patterns that prioritize interoceptive and homeostatic information processing. For example, autobiographical recall of sadness episodes seems to be related to a higher activation of bodily sensations, such as an increased heart rate and systolic and diastolic blood pressures, along with increased breathing frequency and variability.^59^ Indeed, sadness has been directly linked to interoceptive awareness and the process of “embodiment,” which allows individuals to have an integrated sense of the physiological condition and postures of their bodies. For example, it has been shown that individuals with greater interoceptive awareness are more sensitive to expressions of sadness^60^ and that motor performance, as well as the observation of dynamic whole-body expressions of sadness, increases the subjective feeling of sadness in the observer.^61^

Interestingly, happiness was the second emotion that mostly activated brain regions mapping body segments (8.22%), which is on the opposite spectrum compared with sadness. One common characteristic between happiness and sadness is that, both in the self-reported silhouettes of body sensations and in the equivalent maps derived from fMRI data, the two hottest spots were the face and the trunk.

### Discrete emotions overlap with specific-body-segments maps

After observing that all five emotions re-enacted by the participants showed an embodied nature (i.e., overlap with our sensorimotor conjunction map), we explored whether each emotion may engage specific maps of bodily activations, as evidenced in the behavioral studies of Nummenmaa and colleagues.^25^^,^^26^^,^^39^ The results showed that emotions overlapped with the four discrete recreated sensorimotor maps (i.e., face, hands, trunk, and feet) with different involvement of body segments depending on the emotion evoked by the participants.

In particular, the face seemed to be the body segment mostly involved in the feeling of some emotions (i.e., sadness and happiness). A similar activation pattern was also found in the behavioral analysis of the silhouettes colored by the participants after completing the tasks. Specifically, by analyzing the percentage of pixels colored by each participant in the four discrete body segments, we found that emotions were felt in different body districts extensively, similar to the neural data.

The fact that the face was activated by most emotions is not surprising, as the face represents a unique social stimulus that conveys not only emotion perception (e.g., facial expression) but also emotion generation (e.g., the embodiment of emotions). Indeed, temporary inactivation of the brain’s face representation areas with repetitive transcranial magnetic stimulation (rTMS) tends to impair emotion recognition.^62^^,^^63^^,^^64^ Similarly, the right somatosensory cortex appears to be a critical component, along with structures such as the amygdala and right visual cortex, useful in retrieving socially relevant information from faces.^65^

The emotional recall task may have facilitated the mental representation of facial expressions congruent with the episode participants were listening to. For example, during scanning, some participants may have recalled the happy expressions of those who took part in their graduation or experienced sad facial expressions during the re-enactment of grief events. Studies using electromyogram show that imagining negative emotional events are associated with increased activity in the corrugator supercilia, whereas imagined positive emotional events are associated with increased zygomatic major activity.^59^

Nevertheless, in our neural and behavioral data, we cannot observe whether there were specific body signals that seemed crucial to classify a single emotion. This is in line with neuroimaging studies^5^^,^^23^^,^^65^^,^^66^^,^^67^ and behavioral studies^24^^,^^25^^,^^38^^,^^39^^,^^68^ that show that discrete emotions activate the representation of wide-body districts rather than single body parts. Each discrete emotion appears to recruit a set of interacting subcortical and cortical regions that form specialized and distributed neural pathways. The subjective process of feeling emotions is partly grounded in dynamic neural maps representing several aspects of the organism’s continuously changing internal state. In general, the fact that we found activity in somatosensory and motor areas during the emotional recall task, suggests an involvement of somatosensory/motoric codes to the emotional experience. This corroborates the view of a topographical distribution of emotion-related bodily sensations.^24^^,^^25^^,^^26^^,^^38^^,^^39^ Thus, we may speculate that the neural bodily maps, as identified in our study, contribute to the conscious feelings of recalled emotions.

### Dissociation between self-report and brain activity

Despite the possibility of visually observing similar patterns between neuroimaging data and behavioral data, we did not find a direct relationship between neuronal activation (i.e., voxels) during the emotional recall task and the subjective representation of participants' colored silhouettes (i.e., pixels) after scanning. This sounds to be at odds with previous findings, but it could depend on methodological aspects and the nature of the two tasks. Indeed, in the behavioral data analysis, pixels were measured through a pen-and-paper instrument and at a time after the immediate generation of emotions via audio track. Conversely, voxels were measured during audio contingency and in a different experimental scenario. Besides, the dissimilarity between the neural and the behavioral data could be related to the generation of emotions itself, which, on the one hand, was immediate and guided through audio tracks and, on the other hand, was based on mere mnestic effort. Indeed, the measurement of the number of colored pixels for different body segments was done on a purely motor behavior (i.e., participants’ drawings). In contrast, voxel activation reflects a more distributed activity on the cortex, in which the motor system is only one of the aspects considered, and tactile behavior was involved. Future investigations could explore whether there are other brain areas with body representation, in addition to those we have considered, that are activated in a pattern similar to that observed in the behavioral task, using different approaches that may extend our original hypothesis (e.g., whole-brain searchlight Multivariate Pattern Analisys (MVPA). Although we did not observe the same level of specificity in behavioral studies,^24^^,^^25^^,^^26^^,^^38^^,^^39^ the highly specific activity of the sensorimotor system suggests that the affective and emotional processes may also have an embodied component. In support of the involvement of the somatosensory system in different phases of emotional processing, research in which internal bodily representations are manipulated has demonstrated the body’s distinct role in the experience of emotions.^33^^,^^34^^,^^69^^,^^70^^,^^71^^,^^72^^,^^73^ For example, research focused on interoceptive signals (e.g., heart rate) has shown that manipulation of afferent cardiovascular signals can alter the detection and processing of affective stimuli. In particular, baroreceptor activation can facilitate the detection of fear and strengthens the attribution of emotional salience^72^ and can also impact the visual processing of fearful stimuli and contribute to the increased attentional capture of threat signals.

Similarly, studies in clinical populations associated with abnormal emotional regulation (e.g., depressive disorder, post-traumatic stress disorder, anxiety and panic disorders, and obsessive-compulsive disorder) have revealed structural and functional alterations in the somatosensory cortex,^69^^,^^74^^,^^75^^,^^76^^,^^77^ and evidence of reduced somatosensory system recruitment during emotion discrimination has also been found in autistic spectrum disorder (ASD), in which ASD participants are observed to show a selective reduction of SEPs amplitudes (P100) comparing with typically developing participants during a facial emotion discrimination task.^77^ In summary, our study supports embodied approaches to the generation of emotions^11^^,^^12^^,^^13^ by revealing that emotions have a specific neural makeup strongly grounded in sensorimotor processes at the roots of our conscious feelings of emotions.

### Limitations of the study

Our study presents a series of limitations. First, we have only focused on five emotions that cannot fully cover the complexity of emotions we have to deal with in social interactions and also in our inner self. Future studies should investigate if complex and social emotions share a similar brain representation of the body. In addition, individual variables (such as gender or personal traits) may have influenced the body-emotion link. For instance, increased interoceptive sensitivity or a more embodied approach to interpreting and describing emotions is connected with more intense physical, self-reported, and neural experiences while experiencing emotional stimuli.^78^^,^^79^^,^^80^

Furthermore, the present study involved only female participants due to the evidence in the literature indicating that emotional expressions might differ between males and females. Indeed, it is important in affective research (e.g., affective neuroscience) to take into account sex differences. Research indicates that men and women possess different skills related to sending and receiving of emotional messages. For example, it has been shown that women generate facial electromyographic patterns of greater magnitude and report more robust experiences of emotion while imagining emotional situations.^81^ Moreover, sexual differentiation of the human brain can also be observed: hormones, sex chromosome genes, and sex-specific environments have independent parallel differentiating effects that can interact with each other to cause sex differences in the brain.^82^^,^^83^^,^^84^^,^^85^ Therefore, in such a difficult task as recalling emotionally salient episodes in a noisy, non-ecological environment (such as an fMRI), we preferred to recruit participants who seem somehow more advantaged in expressing their emotions. Therefore, it remains to be explored whether this body-emotion relationship can also be generalized to a male population and whether differences can be observed.

Finally, our study cannot make a definitive statement about a causal relationship between the activations of specific parts of the somatosensory maps and the embodied nature of specific emotions. Indeed, the technique used only provides a correlational account of the relationship between emotion generation and specific brain areas. Nevertheless, our study provides insights into a possible relationship by adding a neural perspective to the emergence of the subjective feeling of emotion as associated with bodily sensations. In a recent unpublished study, we have used tACS (transcranial alternating current stimulation) to address the causal role of the sensorimotor cortex in the modulation of perceived emotions. Although these findings cannot be considered conclusive because they are only derived from one study, they nevertheless shed light into the potential role of the sensorimotor system in the generation of emotions.

## STAR★Methods

### Key resources table

**Table:** 

REAGENT or RESOURCE	SOURCE	IDENTIFIER
**Deposited data**		

Script for digitalized silhouettes and Supplementary materials	• OSF repository • Mendeley repository	https://osf.io/wujr4/ https://doi.org/10.17632/xtmpzwyk7f.1

**Software and algorithms**		

3.0 E-Prime	Psychology Software Tools, Pittsburgh, PA	https://pstnet.com/products/e-prime/
MRIcron	NITRC NeuroImaging Tools & Resources Collaboratory	https://www.nitrc.org/projects/mricron
MRIcroGL	NITRC NeuroImaging Tools & Resources Collaboratory	https://www.nitrc.org/projects/mricrogl
MATLAB_R2021b	Math Works, Natick, MA, USA	https://it.mathworks.com/products/matlab.html
Statistical Parametric Mapping (SPM 12)	Wellcome Department of Imaging Neuroscience, London, UK	https://www.fil.ion.ucl.ac.uk/spm/software/download/

### Resource availability

#### Lead contact

Further information and requests for resources should be directed to and will be fulfilled by the lead contact, Michelle Giraud (m.giraud@campus.unimib.it).

#### Materials availability

This study did not generate new unique reagents.

#### Data and code availability

• Data: MATLAB data to digitised pen-and-paper silhouettes have been deposited at Mendeley Dataset and are publicly available as of the date of publication. DOIs are listed in the key resources table.
• Code: All original code for MATLAB script to digitised pen-and-paper silhouettes has been deposited at Mendeley Dataset and is publicly available as of the date of publication. DOIs are listed in the key resources table.
• Any additional information required to reanalyse the data reported in this paper is available from the lead contact upon request.

### Experimental model and study participant details

#### Human participants

Twenty-seven female participants (mean age = 27.5 years old, SD = 5.9, range: 22–50 years old), all Italian and White participated in the experiment. Participants had no neurological or psychiatric history, were right-handed and had normal or corrected-to-normal vision. All gave informed consent prior to testing and were informed about the procedure and potential risks associated with the fMRI scan. The study was conducted in accordance with the Declaration of Helsinki and was approved by the ethical committee of the University of Milano-Bicocca (Protocol number: 0090060/20). One participant was discarded from the analysis due to non-pathogenic intraparenchymal calcification, resulting in twenty-six participants in total. Before testing, all participants answered the Toronto Alexithymia Scale (TAS-20,^86^), which revealed no alexithymia among our sample.

### Method details

#### Experimental paradigm

A few days before fMRI scanning (i.e., 4–5 days before), all participants were instructed on the procedure of the experiment and an experimenter collected their autobiographical episodes. During the interview, participants were asked to put headphones on and listen to an audio-track that mimicked the actual scanning environment. They were asked to think of and tell the researcher about episodes of intense autobiographical emotions involving happiness, serenity, sadness, fear, and anger (3 episodes for each of the five emotions, for a total of 15 episodes), and as many specific but emotionally neutral episodes, considered as a baseline. For both conditions (i.e., emotional, and neutral episodes) participants were encouraged to provide as much detail as possible and to focus on these recalls carefully. No attempt was made to artificially force the participant’s narrative into specific themes, and all participants made the effort to recall the most powerful events associated to each discrete emotion.

Instead, neutral memories consisted of in-depth recollections of unemotional but precise daily actions, such as waking up in the morning, making breakfast, taking the car to work, and so on.

All 30 episodes were noted down during the interview, verified with the participants and then recorded by the researcher into audio tracks that were used on the day of the experiment during the scanning. All auditory stimuli were delivered using the software 3.0 E-Prime (Psychology Software Tools, Pittsburgh, PA) via an MR-compatible screen or headphones.

##### Somatosensory functional localizer

In the tactile stimulation task, the experimenter stimulated participants’ faces, hands, trunk, and feet according to visual instructions. The stimulation was applied using two 60g Von Frey filaments, employed to control for the intensity applied to each tactile stimulation across participants. The tactile stimulation was bilateral and performed four times on each body part, labeled with a pen before participants entered the scanner. The sequence of the stimulated body segments was the same for all participants: hands, feet, face, and trunk (see Figure 4A). The stimulation blocks lasted 20s each and were alternated with resting scans, according to a block design. During the rest baseline conditions, subjects were instructed to relax and avoid any intense thought.

##### Motor functional localizer

During the motor task, participants were asked to perform specific movements of the face, hands, trunk, and feet at a frequency of about 1Hz. Subjects practiced the task before the scanning to ensure that each movement was performed correctly and at the requested pace. The type of movement differed according to body part, and participants received audio instructions regarding which part of the body they had to move during the scanning. Face movements implied stretching the mouth to the left and right side; hand movements were performed by opening and closing the fingers of both hands; feet movements were extension and flexion movements of the toes; trunk movements were contractions of the abdominal muscles (i.e., relaxing and contracting them). During the rest baseline conditions, subjects were instructed to relax and refrain from thinking. Each block was 20s long, followed by 20s of rest. Each condition was repeated four times with a fixed order: hands, feet, face, and trunk (see Figure 4B).

##### Emotional recall task

During the Emotional recall task, participants were requested to mentally recall specific emotional or neutral events and bodily sensations associated with those experiences. The voice of the experimenter guided them. We presented 15 emotional episodes (3 for each emotion) alternated with a neutral episode and presented in random order throughout the session. Each emotional/neutral episode lasted 14s. After the recall of each episode, participants were asked to rate first the intensity and then the vividness of each emotional event by pointing a number between 1 and 5 with their right hand, where 1 indicated the minimum intensity/vividness and 5 the maximum intensity/vividness. The intensity referred to how strongly the emotional feeling was retrieved, while vividness indicated how accurately the episode was retrieved (see Figure 4C).

#### Procedure

The experimental session in MRI was divided into three different fMRI runs, lasting 15 min for the somatosensory and motor functional localizer task and 20 min for the emotional recall task, for a total of 1 h 20 min of scanning. The scanning started with the somatosensory localizer, followed by the emotional recall task, and ended with the motor localizer. A 2-3-min break was allowed to participants between each block, but each subject could ask for a longer break between blocks. The three tasks were performed within the same experimental sessions.

During the prior interview (i.e., 4–5 days before the experiment) and immediately after the MRI scan, participants were presented with a paper version of the emBODY tool (https://version.aalto.fi/gitlab/eglerean/embody,^26^), which consisted of a silhouette of the human body. Through this self-assessment tool, participants were asked to color the different body parts in red or blue, depending on their perception of increased (red) or decreased (blue) activation during the recall of the emotional episode’s recorder by the fMRI scans. Red was used for those parts of the body in which participants felt body activity had become stronger or faster (e.g., higher body temperature, increased muscle movements/tensions, increased internal body signals). Instead, blue was used for those parts of the body in which participants felt body activity had become weaker or slower (e.g., lower body temperature, shivers, freezing sensations, slowing of internal body signals). Therefore, at the end of the whole experiment, we obtained two types of emBODY silhouettes: a pre-scan and a post-scan silhouette.

##### Functional MRI image acquisition procedures

Whole-brain functional images were acquired using a 3.0-T Ingenia scanner (Philips). Gradient-echo T2∗-weighted transverse echo-planar images (EPI) with blood oxygenation level-dependent (BOLD) contrast were acquired (scan parameters: TR = 2000 ms, TE = 30 ms, 35 transversal slices, descending not interleaved acquisition, 4 mm slice thickness, with no interslice gap, FA = 75°, FOV = 240 mm, matrix size = 80 × 80 mm).

For each participant, we collected 325 volumes for the somatosensory localizer task, 325 volumes for the motor localizer task, and 590 volumes for the emotional recall task (for a total of 1285 functional volumes) plus a high-resolution T1-weighted anatomical scan (TR = 2250 ms, TE = 2.6 ms, 192 sagittal slices, voxel size 1 × 1 mm, FA = 9°, Inversion Time (TI) = 900 ms). The first 15 volumes of each sequence, corresponding to the task instructions, were discarded from the fMRI analysis.

##### Pre-processing & Statistical Parametric Mapping

After image reconstruction, raw data visualization and conversion from DICOM were performed using MRIConvert (lcni.uoregon.edu). All subsequent data analysis was performed in MATLAB_R2019b (Math Works, Natick, MA, USA), using Statistical Parametric Mapping (SPM12, Wellcome Department of Imaging Neuroscience, London, UK).

Functional images were realigned to the first acquired volume and unwrapped to minimize the effect of the subject’s movement during the session. The high-resolution T1-weighted structural image of each participant was segmented and normalized to the MNI (Montreal Neurological Institute) stereotactic space to allow between-subject comparison (Ashburner and Friston, 2005), and it was then co-registered to the realigned and unwrapped functional volumes. The functional images were normalized by applying the Deformation Fields employed during the structural data segmentation. The data matrix was interpolated to produce 2 × 2 × 2 mm voxels. The normalized scans were finally smoothed using a Gaussian filter with 10 × 10 × 10 mm as the full width at half-maximum (FWHM) value to improve the signal-to-noise ratio in the data, a smoothing level optimal for correction for multiple comparisons.^87^

### Quantification and statistical analysis

#### Statistical analysis of the behavioral data

##### emBODY task (paper-and-pencil version)

Subject-wise bodily sensation maps were digitized and then pre-processed using an analysis stream implemented in MATLAB (version R2021b) using the Statistics and Machine Learning Toolbox (version 12.2, The MathWorks, Inc., Natick, Massachusetts, United States) and Image Processing Toolbox (version 11.4, The MathWorks, Inc., Natick, Massachusetts, United States). The pre-processing steps followed a similar approach described in previous studies.^26^^,^^69^^,^^88^

The pre and post pen-and-paper silhouettes were scanned with a professional scanner (device model: MX-M754N) at a resolution of 3003×00 dpi in color. All emotion-wise body maps were normalised through alignment by registration to a standard body template using the Mattes mutual information metric configuration, transforming the 2-D images so that it is registered with the reference image (i.e., template image).^89^ The alignments of the body map to the template were visually inspected, and there was no need to perform any correction. Pixels outside the body’s boundaries were discarded (see Figure S3, and Mendeley Data: https://doi.org/10.17632/xtmpzwyk7f.1 for MATLAB scripts). Activation and deactivation maps were represented by 50.365 pixels each. Uncoloured pixels were coded as 0, while colored pixels were coded as 1. In addition, to facilitate comparison with,^26^ Figure 2 displays the pixel-wise activation of the body map following a similar approach as described in the referenced study. The same analyses were performed for pre- and post-scan silhouettes, thus obtaining for both silhouettes a proportion of activations per single emotion within each body part. The two data were then correlated to assess the reliability between participants' pre- and post-subjective judgments.

##### The subjective experience of emotions: Participants' ratings and single-subject level analysis

At the end of the audio track of each emotional and neutral episode, participants were asked to indicate with the fingers of their right hand the intensity and vividness of the episode they had just heard (i.e., from 1 to 5). The ratings were collected and analyzed to confirm whether there was a difference between the emotional and neutral episodes and used as a regressor in the fMRI analyses. Moreover, we analyzed the emBODY silhouette of each participant by dividing them into four discrete body parts (i.e., the same one selected during the scanning: face, hands, trunk, and feet).

For each body part, we calculated the ratio between colored pixels and the total number of pixels within that specific body part. For each subject, we then summed the percentage obtained from each body part. The percentage of activation from each body segment was combined into a single body map by summing pixel-wise activation (see Figure 1A). This provided us with the total percentage of activation of a specific body segment colored by the participants after the scanning.

#### Statistical analysis of the fMRI data

##### Somatosensory and motor localizer tasks

Global differences in the fMRI signal were removed from all voxels with proportional scaling. High-pass filtering (128 s) was used to remove artifacts to the fMRI signal, such as physiological noise from cardiac and respiratory cycles.

First, a fixed-effect block analysis was performed on each participant (first-level analysis) to characterize the BOLD response associated with each tactile and motor task compared to rest. The different conditions were modeled as blocks using a boxcar regressor and then convolved with the canonical hemodynamic response function in order to model the predicted BOLD signal.^90^ We created a contrast image for each effect of interest (four contrast images for each task, for a total of eight contrast images for each subject). For example, for the motor localizer task, we created the following contrast images: Hands Movement > Rest; Feet Movement > Rest; Trunk Movement > Rest; Face Movement > Rest.

To permit generalization to the population level using group-based statistical inference, the individual contrast images generated by the fixed-effect analyses were entered in a second-level ANOVA, conforming to random effect analyses.^91^^,^^92^

We assessed the following effects:

(1) Across-body segment effect: we first evaluated the brain activations shared by all body segments, for both the motor and the functional localizer task, as a single conjunction effect.^91^^,^^93^
(2) Specific-body segment effects: we calculated the specific effects for each body segment in the tactile and motor tasks (e.g., specific hand effect: hands movements & hand tactile stimulation > all the other body segments movements & tactile stimulation).

The voxel-wise threshold applied to the statistical maps before the cluster’s correction was *p* < 0.001 uncorrected, as recommended by.^87^ For clusters significant at the *p* < 0.05 FWER-corrected level, we also report the other peaks at *p* < 0.001.

##### Emotional recall task

First, we performed a first-level analysis using the same approach described for the somatosensory and motor localizer tasks. For each participant, we performed a fixed-effect block analysis to characterize the BOLD response associated with recalling emotionally salient events, as opposed to recalling neutral events. The different conditions were modeled as blocks using a boxcar regressor and then convolved with the canonical hemodynamic response function in order to model the predicted BOLD signal.^90^ We modeled the BOLD activity related to the intensity/vividness assessment as a possible confounding variable.

We created a contrast image for each effect of interest, for a total of five contrast images for each subject: happiness recall > neutral recall; sadness recall > neutral recall; anger recall > neutral recall; serenity recall > neutral recall; fear recall > neutral recall.

###### Group analysis (across subjects)

To permit generalization to the population level using group-based statistical inference, the individual contrast images generated by the fixed-effect analyses were entered in a second-level one-way ANOVA conforming to random effect analyses.^91^^,^^92^

We assessed the following effects:

(1) We evaluated the brain activity evoked by the emotional recall task compared to the neutral episodes (i.e., the main effect of emotional recall: All emotions > all neutral episodes).
(2) We assessed the specific effects of each emotion of the emotional recall task (e.g., specific Happiness effect: Happiness recall > neutral recall) (see Figure S4).
(3) We calculated to what extent emotions are generally embodied by masking each emotion’s brain map with the conjunction maps obtained in the tactile and motor localizer tasks (e.g., the embodiment of happiness: Happiness recall>neutral recall masked by sensorimotor conjunction map).
(4) We estimated to what extent emotions are specifically embodied by masking each emotion with the discrete body-segment maps obtained in the tactile and motor localizer tasks (e.g., the body segment specificity for happiness: Happiness recall>neutral recall masked by sensorimotor discrete body-segment maps).

The voxel-wise threshold applied to the statistical maps before the cluster-wise correction was *p* < 0.001 uncorrected, as recommended by^87^). For clusters significant at the *p* < 0.05 FWER-corrected level, we also report the other peaks at *p* < 0.001.

###### Single-subject level analysis

To define a similar description for the fMRI data that could take into account individual responses and intersubjective variability, we calculated individual emotion-specific activation patterns at the first level and masked these effects on the body-specific group-level sensorimotor maps.

We assessed the following effects:

(1) We estimated the subjective feeling of emotions by assessing one each participant at a time the effect of individual emotions (i.e., happiness, serenity, fear, anger, and sadness) and masking it on the discrete body segment maps obtained in the tactile and motor localization tasks (e.g., happiness>neutral recall masked by discrete sensorimotor body segment maps at the single-subject level).
(2) We evaluated the possible congruence between body distribution of emotions as depicted by self-reports and fMRI: we created five silhouettes, one for each emotion, and divided them into the four body parts considered in this study, thus obtaining silhouettes similar to those digitized by the emBODY task.

The voxel-wise threshold applied to the statistical maps before cluster correction was *p* < 0.05 uncorrected to maximize the chance of detecting effects in the less sensitive single-subject fixed-effect analyses.

## Supplemental information

Figure S1. Qualitative comparison with the results reported in Nummenmaa et al. (2014), related to paragraph "The subjective experience of emotion: Single-subject analysis on self-report body silhouettes" in the Results section (A) Nummenmaa et al. (2014) bodily maps of emotions. The warm colours indicate increasing activation, while the cool colours represent decreasing activation. The colour bar shows the t-statistic range. (B) Silhouettes created from the participants’ self-report: warm colours indicate increasing activation, while cool colours represent decreasing activation. The colour bar shows the t-statistic range.

Figure S2. Representational similarity analysis, related to paragraph "Congruence between body distribution of emotions as depicted by self-reports and fMRI" in the Results section (A) Each cell corresponds to the difference between the percentage of activated voxel within each cortically represented body segment and each emotion (e.g., activation of happiness within the hand map minus activation of activation of happiness within the face map), overall obtaining a 20 × 20 matrix. (B) Each cell corresponds to the difference between the percentage of coloured pixel within each body segment of the silhouette and each emotion, overall obtaining a 20 × 20 matrix (All correlations were not significant, *p* = 0.20).

Figure S3. Digitized procedure for self-reports silhouettes, related to paragraph entitled “emBODY Task” in the STAR METHODS Graphic explanation of how the digitization of participants' self-reported silhouettes took place. (A) Digitized body maps were aligned by registration to a standard body template. (B) Pixels outside the body's boundaries were discarded. Uncoloured pixels were coded as 0 (i.e., green), while coloured pixels were coded as 1 (i.e., yellow). (C) The facial, hands, trunk, and feet activation were analysed separately. The percentage of activation for each body segment was computed and combined by summing the activity of the two maps.

Figure S4. Contrast map of each individual emotional recall task and neutral autobiographical episodes, related to paragraph entitled “Emotional recall task” in the STAR METHODS section (A) Brain regions that resulted significantly active for the contrast “Happiness episodes recall > Neutral episode recall”. (B) Brain regions that resulted significantly active for the contrast “Serenity episodes recall > Neutral episode recall”. (C) Brain regions that resulted significantly active for the contrast “Anger episodes recall > Neutral episode recall”. (D) Brain regions that resulted significantly active for the contrast “The figure illustrates the simple effect of the contrast” Fear episodes recall > Neutral episode recall. (E) Brain regions that resulted significantly active for the contrast “Sadness episodes recall > Neutral episode recall”. All the data are reported by applying the same statistical threshold reported in the tables and discussed in the text (puncorr < .001 at the voxel level and pFWER-corr < .05 at the cluster level).

Table S1. Participants’ ratings for each emotion, related to paragraph "The subjective experience of emotions: Single-subject analysis on participants’ ratings" in the Results section

Table S2. Conjunction map of motor execution and tactile stimulation (x, y, and z values express the millimetric distance from the origin in the MNI stereotactic coordinate system), related to Figure 2A

Table S3. Body-segment-specific maps (x, y, and z values express the millimetric distance from the origin in the MNI stereotactic coordinate system), related to Figure 2B

Table S4. Emotional recall > Neutral recall (x, y, and z values express the millimetric distance from the origin in the MNI stereotactic coordinate system), related to Figure 3A

Table S5. Neutral recall > Emotion recall (x, y, and z values express the millimetric distance from the origin in the MNI stereotactic coordinate system), related to Figure 3B

Table S6. Discrete emotions masked over the whole-body sensorimotor maps (x, y, and z values express the millimetric distance from the origin in the MNI stereotactic coordinate system), related to Figure 5

Table S7. Happiness emotions masked over body-segment-specific maps (x, y, and z values express the millimetric distance from the origin in the MNI stereotactic coordinate system), related to Figure 6

Table S8. Serenity masked over body-segment-specific maps (x, y, and z values express the millimetric distance from the origin in the MNI stereotactic coordinate system), related to Figure 6

Table S9. Anger masked over body-segment-specific maps (x, y, and z values express the millimetric distance from the origin in the MNI stereotactic coordinate system), related to Figure 6

Table S10. Fear masked over body-segment-specific maps (x, y, and z values express the millimetric distance from the origin in the MNI stereotactic coordinate system), related to Figure 6

Table S11. Sadness masked over body-segment-specific maps (x, y, and z values express the millimetric distance from the origin in the MNI stereotactic coordinate system), related to Figure 6
