# Supplementary material for: Mapping the emotional homunculus with fMRI
Source: iScience. 2024 May 18;27(6):109985. doi: 10.1016/j.isci.2024.109985 (PMC11167434; doi:10.1016/j.isci.2024.109985)
Supplement: Figure S1. Qualitative comparison with the results reported in Nummenmaa et al. (2014), related to paragraph "The subjective experience of emotion: Single-subject analysis on self-report body silhouettes" in the Results section — (A) Nummenmaa et al. (2014) bodily maps of emotions. The warm colours indicate increasing activation, while the cool colours represent decreasing activation. The colour bar shows the t-statistic range. (B) Silhouettes created from the participants’ self-report: warm colours indicate increasing activation, while cool colours represent decreasing activation. The colour bar shows the t-statistic range. [file mmc1.pdf]

iScience, Volume *27*

Supplemental information

Mapping the emotional homunculus with fMRI

Michelle Giraud, Laura Zapparoli, Gianpaolo Basso, Marco Petilli, Eraldo Paulesu, and Elena Nava

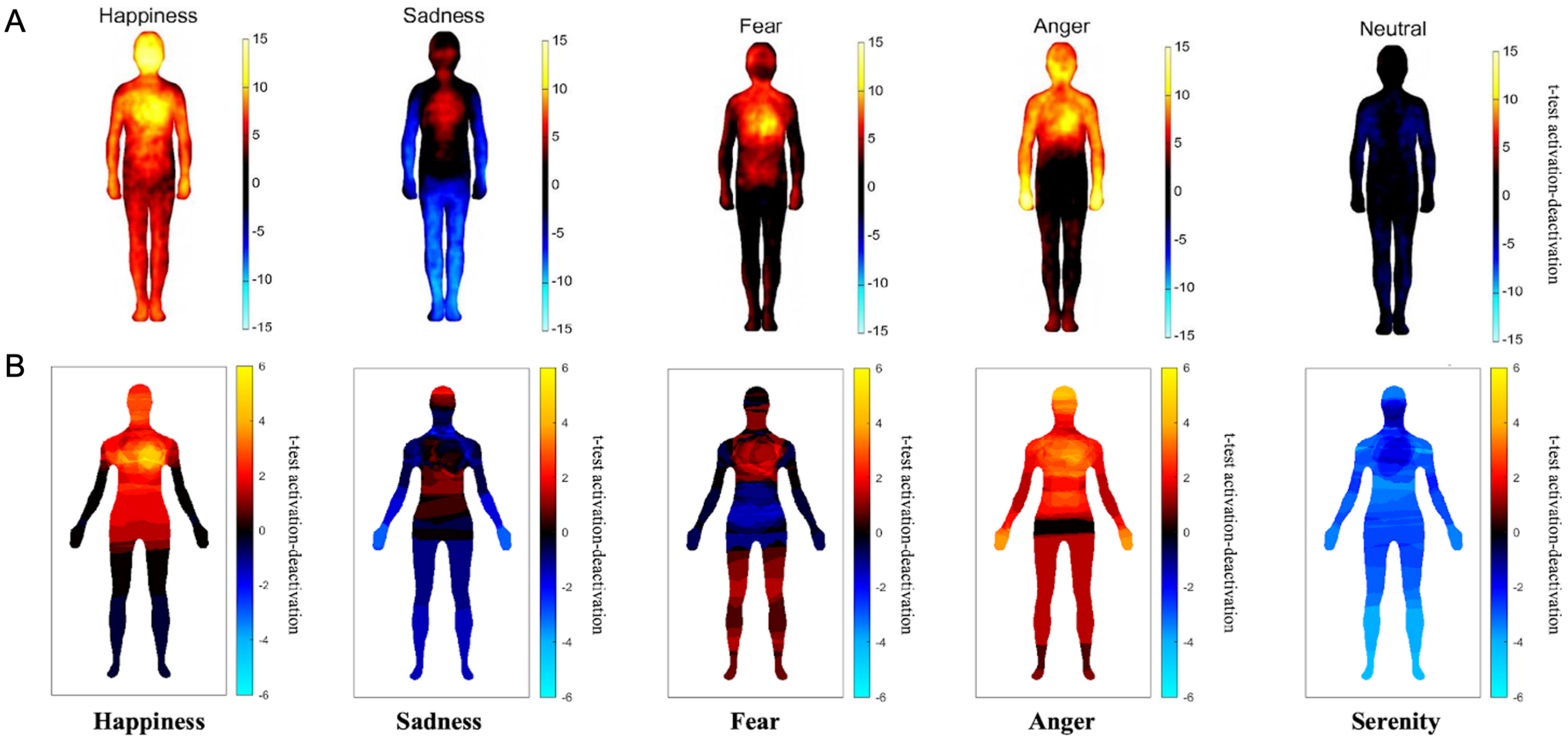

A
Happiness
Sadness
Fear
Anger
Neutral
15
10
5
0
-5
-10
-15
t-test activation-deactivation
B
Happiness
Sadness
Fear
Anger
Serenity
6
4
2
0
-2
-4
-6
t-test activation-deactivation
