# Supplementary figures and images for: Mapping the emotional homunculus with fMRI
Source: iScience. 2024 May 18;27(6):109985. doi: 10.1016/j.isci.2024.109985 (PMC11167434; doi:10.1016/j.isci.2024.109985)

**A**

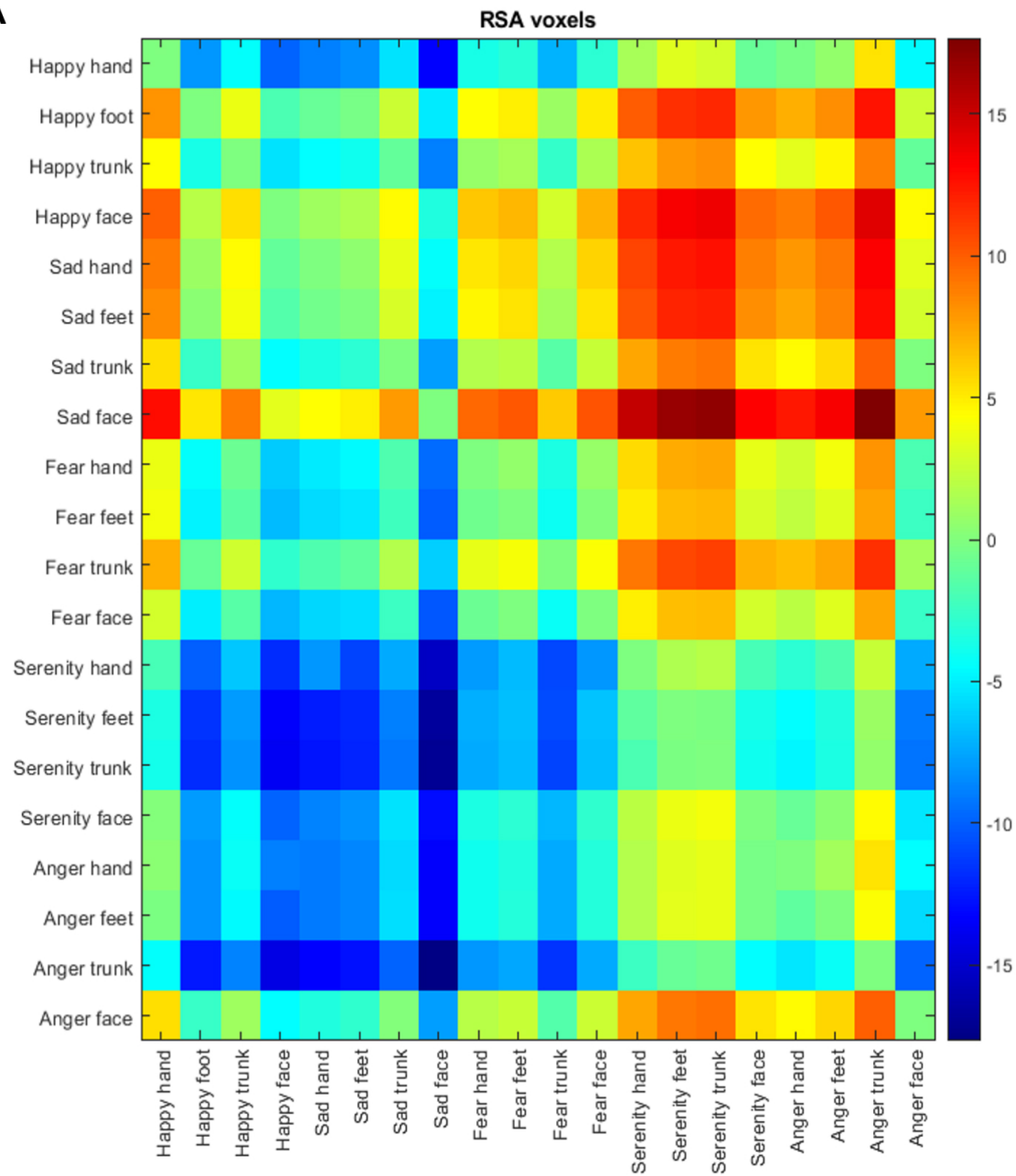

**B**

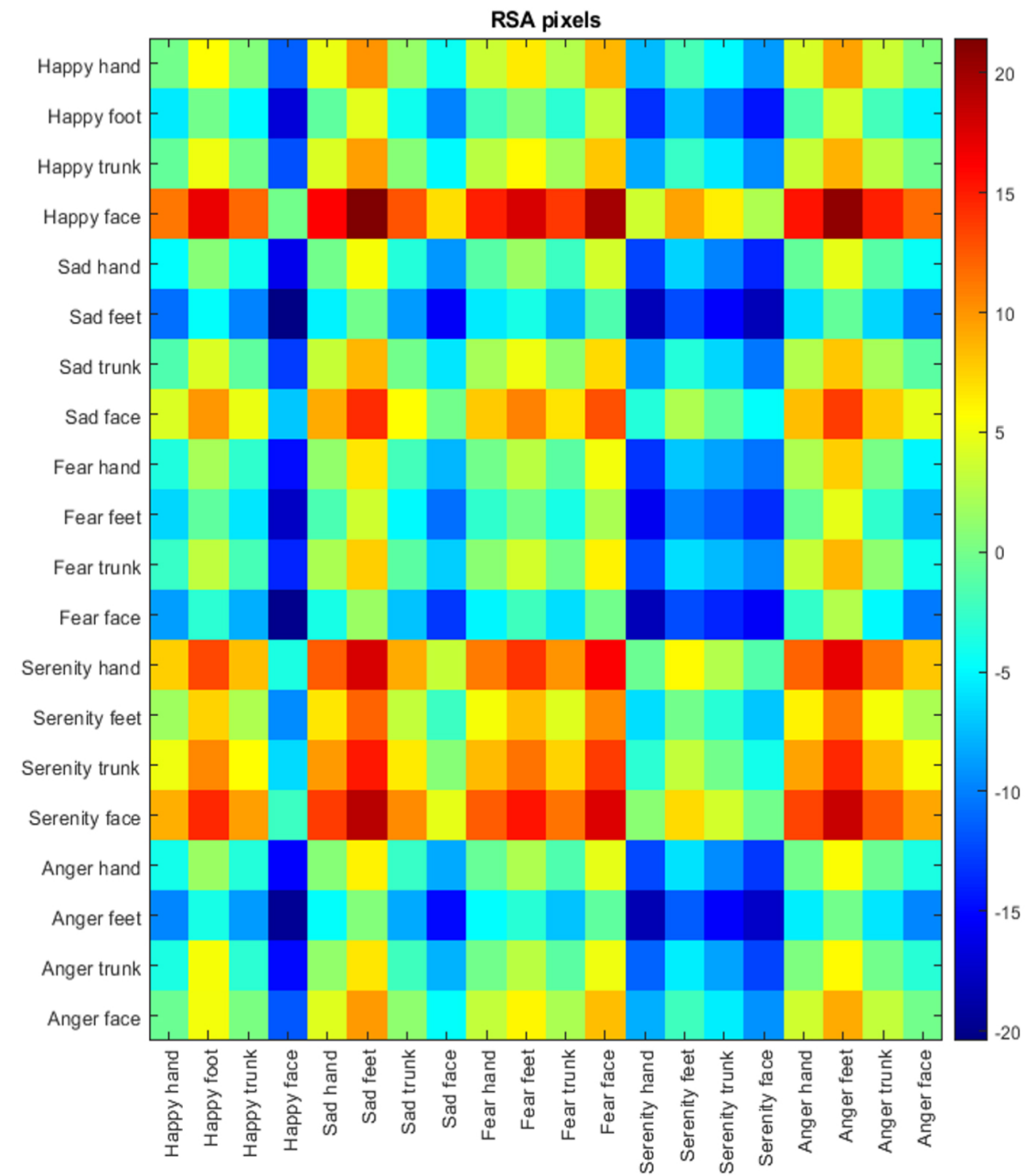

Supplement: Figure S2. Representational similarity analysis, related to paragraph "Congruence between body distribution of emotions as depicted by self-reports and fMRI" in the Results section — (A) Each cell corresponds to the difference between the percentage of activated voxel within each cortically represented body segment and each emotion (e.g., activation of happiness within the hand map minus activation of activation of happiness within the face map), overall obtaining a 20 × 20 matrix. (B) Each cell corresponds to the difference between the percentage of coloured pixel within each body segment of the silhouette and each emotion, overall obtaining a 20 × 20 matrix (All correlations were not significant, p = 0.20). [file mmc2.pdf]

A

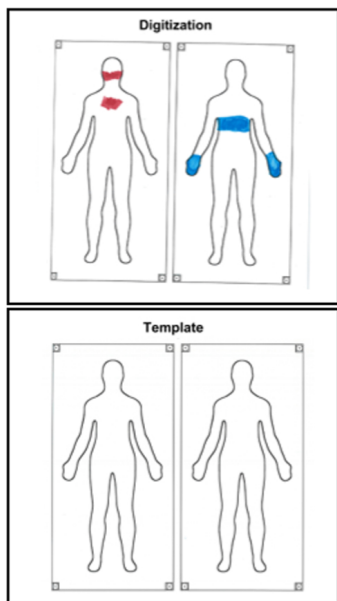

B

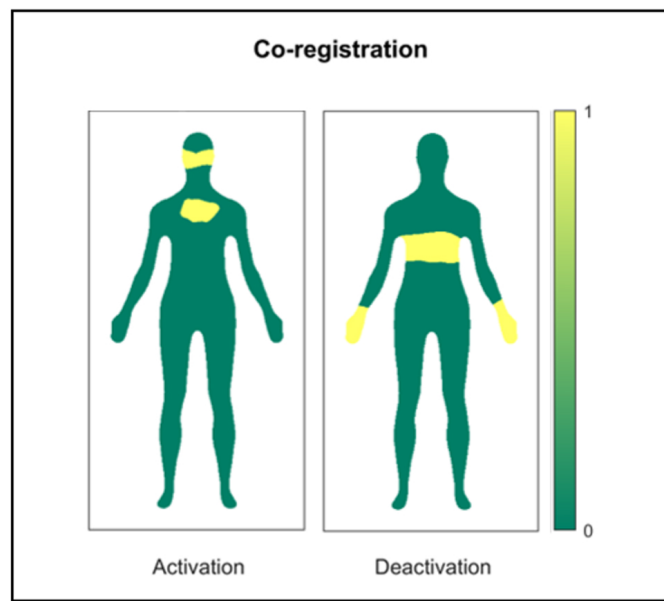

C

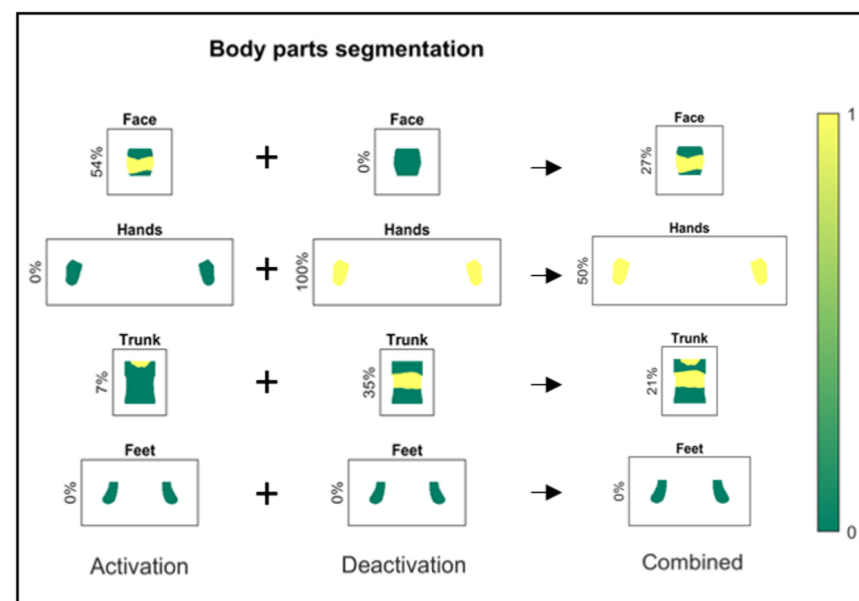

Supplement: Figure S3. Digitized procedure for self-reports silhouettes, related to paragraph entitled “emBODY Task” in the STAR METHODS — Graphic explanation of how the digitization of participants' self-reported silhouettes took place. (A) Digitized body maps were aligned by registration to a standard body template. (B) Pixels outside the body's boundaries were discarded. Uncoloured pixels were coded as 0 (i.e., green), while coloured pixels were coded as 1 (i.e., yellow). (C) The facial, hands, trunk, and feet activation were analysed separately. The percentage of activation for each body segment was computed and combined by summing the activity of the two maps. [file mmc3.pdf]

A)

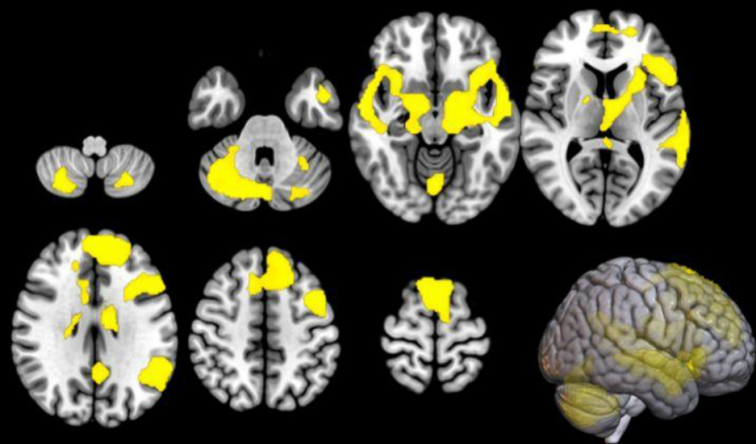

B)

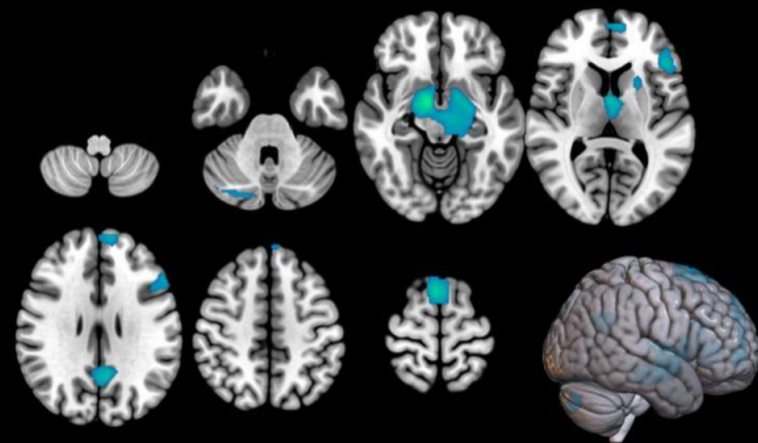

C)

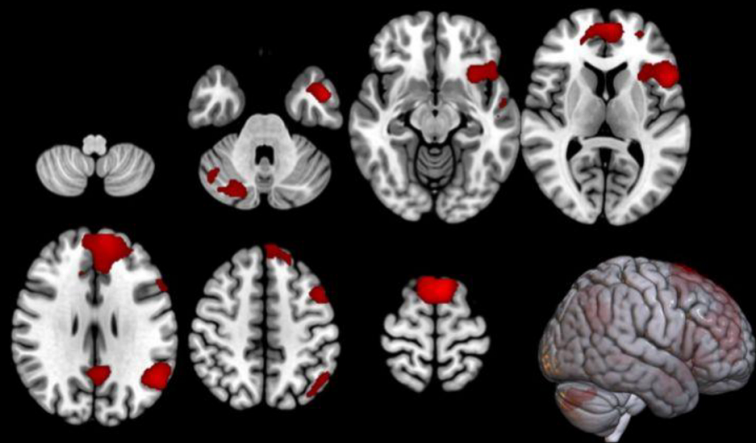

D)

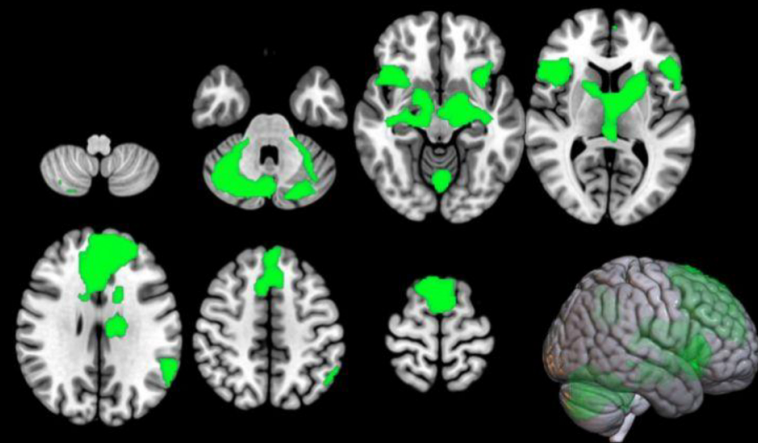

E)

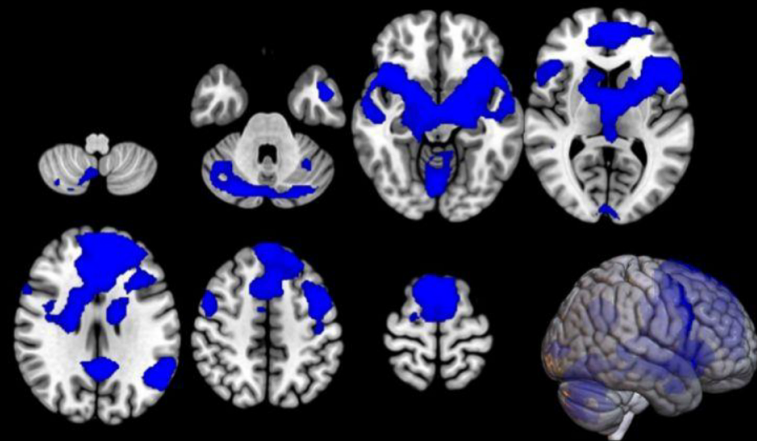

Supplement: Figure S4. Contrast map of each individual emotional recall task and neutral autobiographical episodes, related to paragraph entitled “Emotional recall task” in the STAR METHODS section — (A) Brain regions that resulted significantly active for the contrast “Happiness episodes recall > Neutral episode recall”. (B) Brain regions that resulted significantly active for the contrast “Serenity episodes recall > Neutral episode recall”. (C) Brain regions that resulted significantly active for the contrast “Anger episodes recall > Neutral episode recall”. (D) Brain regions that resulted significantly active for the contrast “The figure illustrates the simple effect of the contrast” Fear episodes recall > Neutral episode recall. (E) Brain regions that resulted significantly active for the contrast “Sadness episodes recall > Neutral episode recall”. All the data are reported by applying the same statistical threshold reported in the tables and discussed in the text (puncorr < .001 at the voxel level and pFWER-corr < .05 at the cluster level). [file mmc4.pdf]
